# Germline and Somatic Pharmacogenomics to Refine Rectal Cancer Patients Selection for Neo-Adjuvant Chemoradiotherapy

**DOI:** 10.3389/fphar.2020.00897

**Published:** 2020-06-17

**Authors:** Elena De Mattia, Rossana Roncato, Elisa Palazzari, Giuseppe Toffoli, Erika Cecchin

**Affiliations:** ^1^Clinical and Experimental Pharmacology, Centro di Riferimento Oncologico di Aviano (CRO), IRCCS, Aviano, Italy; ^2^Radiation Oncology, Centro di Riferimento Oncologico di Aviano (CRO) IRCCS, Aviano, Italy

**Keywords:** pharmacogenomics, rectal cancer, neo-adjuvant chemoradiotherapy, germline, somatic, polymorphism, mutation

## Abstract

Neoadjuvant chemoradiotherapy (nCRT) followed by radical surgery is the standard of care for patients with Locally Advanced Rectal Cancer (LARC). Current selection for nCRT is based on clinical criteria regardless of any molecular marker. Pharmacogenomics may be a useful strategy to personalize and optimize nCRT in LARC. This review aims to summarize the most recent and relevant findings about the role of germline and somatic pharmacogenomics in the prediction of nCRT outcome in patients with LARC, discussing the state of the art of their application in the clinical practice. A systematic literature search of the PubMed database was completed to identify relevant English-language papers published up to January 2020. The chemotherapeutic backbone of nCRT is represented by fluoropyrimidines, mainly metabolized by DPD (Dihydro-Pyrimidine Dehydrogenase, *DPYD*). The clinical impact of testing DPYD*2A, DPYD*13, *c.2846A > T* and *c.1236G > A-HapB3* before a fluoropyrimidines administration to increase treatment safety is widely acknowledged. Other relevant target genes are *TYMS* (Thymidylate Synthase) and *MTHFR* (Methylene-Tetrahydro-Folate Reductase), whose polymorphisms were mainly studied as potential markers of treatment efficacy in LARC. A pivotal role of a *TYMS* polymorphism in the gene promoter region (*rs34743033*) was reported and was pioneeringly used to guide nCRT treatment in a phase II study. The pharmacogenomic analysis of other pathways mostly involved in the cellular response to radiation damage, as the DNA repair and the activation of the inflammatory cascade, provided less consistent results. A high rate of somatic mutation in genes belonging to PI3K (Phosphatidyl-Inositol 3-Kinase) and MAPK (Mitogen-Activated Protein Kinase) pathways, as *BRAF (*V-raf murine sarcoma viral oncogene homolog B1)*, KRAS* (Kirsten Rat Sarcoma viral oncogene homolog)*, NRAS* (Neuroblastoma RAS viral (v-ras) oncogene homolog), *PIK3CA* (Phosphatidyl-Inositol-4,5-bisphosphate 3-Kinase, Catalytic Subunit Alpha), as well as *TP53* (Tumor Protein 53) was reported in LARC. Their pharmacogenomic role, already defined in colorectal cancer, is under investigation in LARC with promising results concerning specific somatic mutations in *KRAS* and *TP53*, as predictors of tumor response and prognosis. The availability of circulating tumor DNA in plasma may also represent an opportunity to monitor somatic mutations in course of therapy.

## Neo-Adjuvant Therapeutic Approaches in Locally Advanced Rectal Cancer

The standard of care for stage II/III Locally Advanced Rectal Cancer (LARC) is neo-adjuvant chemoradiotherapy (nCRT) followed by radical surgery including total mesorectal excision, with the possibility of an adjuvant chemotherapy. This program is based on a long-course radiotherapy (RT, 25-28 fractions in 5-6 weeks) and concomitant chemotherapy including fluoropyrimidines (FP), mainly capecitabine, administered daily for the whole course of treatment.

Despite the progress in the local disease control due to the introduction of nCRT in the treatment of patients with LARC, still there is a considerable proportion of patients (20%) that could be defined as non-responders, showing only minimal tumor response or poor prognosis mainly due to early progression. Another 40% of patients have a partial response and only a variable fraction of 8%–30% of patients achieve a pathological complete response at the time of surgery. The response to nCRT correlates with long-term patients' outcome considering disease-free survival (DFS) and overall survival (OS). Moreover patients achieving a major or complete clinical response after nCRT may be considered for an organ preservation strategy, (i.e., Local Excision, or Wait&Watch) and could avoid an adjuvant treatment (Van Der Valk et al., 2018). Furthermore, nCRT is not devoid of the risk of adverse drug reactions that could in some cases interfere with the treatment plans impacting not only patients' safety and quality of life, but also the success of the anti-tumor treatment.

In the clinical practice it is widely known the crucial importance of the risk stratification on patients. In order to reduce toxicity and improve the patients quality of life, a new generation of studies proposed risk adapted strategies including intensified programs in high risk patients and the possibility to avoid nCRT in low risk patients. Several trials were previously conducted in order to clarify the potential benefit of adopting a chemotherapy association regimen combining FP to other agents, as oxaliplatin or irinotecan (Valentini et al., 2019), or to anti-epidermal growth factor receptor (EGFR) monoclonal antibodies as cetuximab. On the other hand, nCRT intensification including a higher dose of RT appears as a promising approach (Burbach et al., 2014). More recently, Total Neoadjuvant Therapy strategies are hypothesised for high-risk sub-cohorts of patients. These programs are based on the integration of a neoadjuvant chemotherapy consisting in the administration of two or three different drugs, before or after the nCRT. The rationale for this strategy comes from the failure of adjuvant chemotherapy programs caused in most studies by the poor compliance of patients.

The selection of patients for these personalized treatment strategies is currently based essentially on clinical-pathological criteria, including tumor size, clinical T and N stages, distance of tumor from the anal verge, and interval from nCRT to surgery. In particular, some pathological features as circumferential tumor, tumor differentiation, mucinous histology and the presence of macroscopic ulceration, being associated with poor response to nCRT (Hur et al., 2011; Bitterman et al., 2015; Mccawley et al., 2016), demonstrated a detrimental effect in the neo-adjuvant treatment of LARC. Recently, the patients' stratification was shown to be improved using nomograms integrating clinical and radiomic features that may be also extracted from daily images acquired for image-guided RT (Dinapoli et al., 2018; Pirrone et al., 2019). However, despite the promising results, it would be necessary to deepen the knowledge on this topic, by conducting studies on larger datasets, involving a wider range of MRI texture features to enhance the predictive value of these parameters, with further requirement of independent validation to improve the test sensitivity and specificity.

In this context, pharmacogenomics may be a useful strategy to personalize and optimize nCRT in patients with LARC (Agostini et al., 2014; De Mattia et al., 2015; Cecchin et al., 2018). The outcome of an anti-cancer treatment may depend on two genomes: the patient's germline and the tumor cell genomes. The genetic features of the cancer cells are generally related to disease aggressiveness and sensitivity to treatment, whereas the germline genetic variation can mostly impact drugs pharmacokinetics and pharmacodynamics. Consequently, this led to the hypothesis that some patients have germline polymorphisms or somatic mutations in genes encoding for protein with a major role in the nCRT LARC treatment as drug-target, drug-metabolizing enzymes, DNA-repair enzymes, and others that may affect response and safety profile of chemotherapy and radiotherapy in LARC. In this review, we will summarize recent advances in tissue and blood-based pharmacogenomic biomarkers for predicting nCRT response and toxicity in patients with LARC.

By a systematic literature search we reviewed the available pharmacogenomic studies that attempted to identify the role of germline and somatic pharmacogenomics in predicting chemotherapy and radiotherapy based nCRT outcome in patients with LARC.

## Materials and Methods

We performed a systematic literature search on January 2020 using the PubMed Medline Database and the combinations of the following terms: (chemotherapy OR radiotherapy OR chemoradiotherapy) AND “rectal cancer” AND (polymorphism* OR pharmacogenetic* OR pharmacogenomic* OR mutation*) AND (toxicity OR recurrence OR relapse OR survival OR progression). The literature search was completed by adding other articles that were identified with a hand search of the references of relevant works. As inclusion criteria the studies had to be published in English in a peer-reviewed journal. All the papers obtained by the PubMed search were reviewed for those specifically assessing the role on host and somatic genetic markers in predicting the clinical response in patients with LARC receiving pre-operative RT combined with FP-based chemotherapy, with or without other drugs (e.g., oxaliplatin, irinotecan, cetuximab). Eventually, nine and forty-two studies, addressing the host genetic profile's ability to predict the toxicity (**Supplementary Table 1**) and efficacy (**Supplementary Table 2**) of nCRT in patients with LARC, respectively, were selected. With regard to these specific papers, the attention was focused on details (**Supplementary Tables 1** and **2**) concerning the pharmacogenomic panel analyzed, the study population (e.g., sample size, ethnicity) and therapy (e.g., dose and schedule) characteristics, the clinical end-points evaluated and the adopted scoring system for toxicity or Tumor Regression Grade (TRG) classification, along with the main findings (e.g., statistical results). Additional fifteen works concerning the involvement of somatic mutations in determining the nCRT outcome in patients with LARC were selected and discussed in the manuscript. Seven out of these works, published between 2010 and 2012, are aggregated in a meta-analysis (Clancy et al., 2013) to which we referred in the text.

## Neo-Adjuvant Radio-Chemotherapy Combination Treatment in LARC and Pharmacogenetic Markers of Toxicity

**Figure 1:**
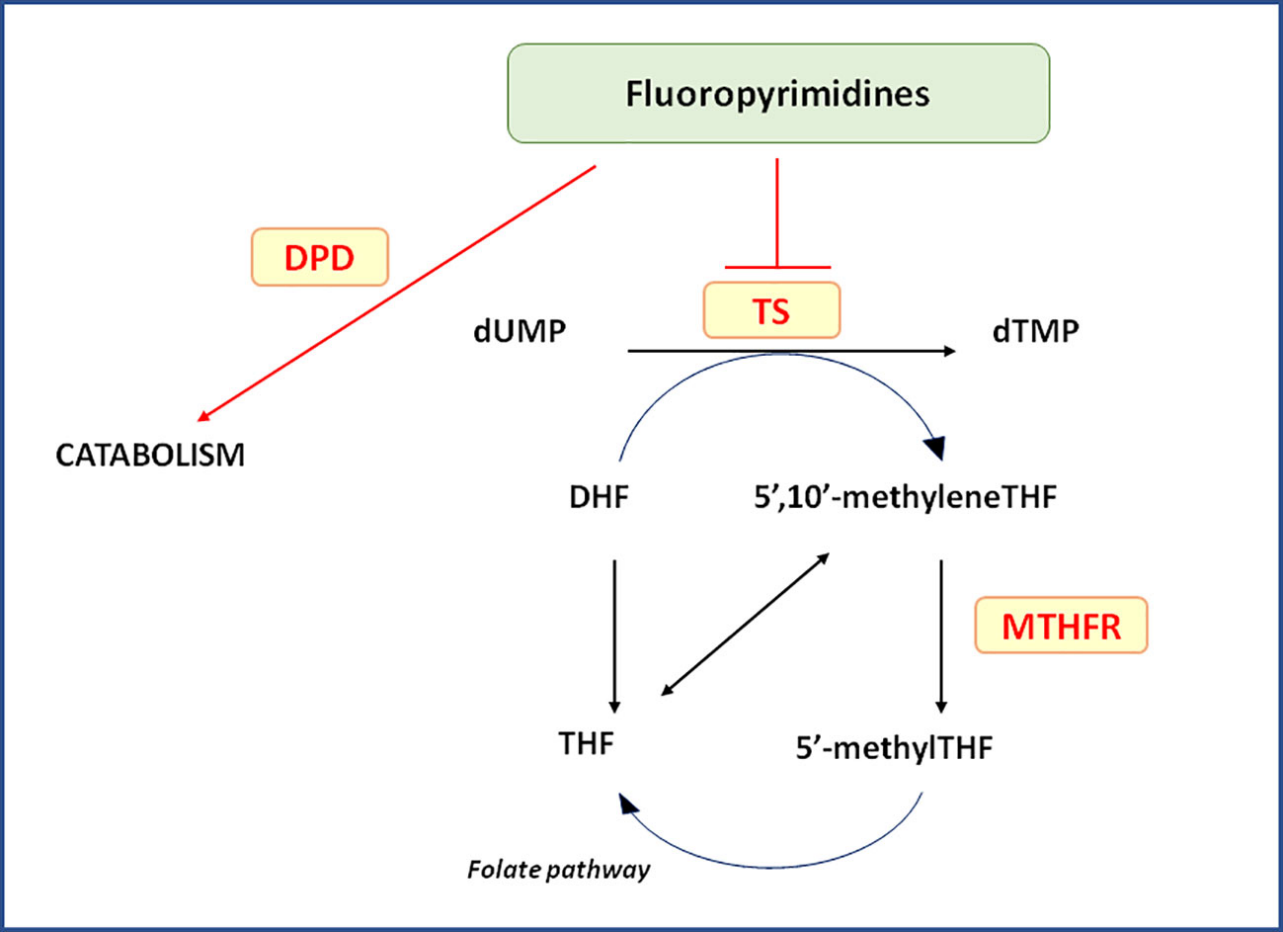
Relevant pathways for pharmacogenetics of Fluoropyrimidines. In the Figure are pictured the most relevant proteins cited in the text and related to the clinical outcome of locally advanced rectal cancer patients receiving neo-adjuvant chemoradiotherapy. 5’-methylTHF, 5-methyltetrahydrofolate; 5’,10’-methyleneTHF, 5,10-methylentetrahydrofolate; DHF, dihydrofolate; DPD, dihydropyrimidine dehydrogenase; dTMP, deoxythymidine 5’-monophosphate; dUMP, deoxyuridine 5’-monophosphate; MTHFR, 5,10-methylenetetrahydrofolate reductase; THF, tetrahydrofolate; TS, Thymidylate synthase.

FP-based nCRT is the standard backbone chemotherapy for pre-operative treatment of patients with LARC. FP exert their anti-tumor action as antimetabolite drugs through different mechanisms including impairment of thymidylate synthase (TS) activity, RNA synthesis and function leading to DNA strand breakage. FP are largely used in the treatment of several malignancies demonstrating a significant improvement in the patients' survival. However, these drugs present a narrow therapeutic index and around 15%–30% of patients suffer from severe toxicity such as diarrhea, nausea, mucositis, stomatitis, myelosuppression, neurotoxicity, and hand-foot syndrome, depending on the treatment regimen. These side effects lead to mortality in approximately 0.5%-1% of patients following 5-fluorouracil (5-FU) and capecitabine monotherapy, respectively (Lunenburg et al., 2016) (**Figure 1**).

FP are used in the neo-adjuvant treatment to increase the radiotherapy cytotoxic effect: this association can exacerbate the toxicity related to the chemotherapeutic treatment. The focused-damaging cytotoxic effect of radiotherapy is exerted on cell structures, proteins, and DNA of tumor cells. Particularly, the DNA is the main target of ionizing radiation. DNA structure is altered by the direct damage of radiation, that causes both single- and double-strand breaks in the DNA molecule, and by the indirect damage of generated free-radicals. The surrounding normal tissue is commonly also affected, resulting mainly in radiation-related intestinal injury, including acute inflammation and late fibrosis. Other radiation-induced early side effects include mucositis, vomiting, diarrhea, cystitis, perineal dermatitis, and hematological dysfunction. Bowel dysfunction, fecal incontinence, bleeding, perforation, genitourinary dysfunction, and pelvic fractures constitute most of the late toxicity (Joye and Haustermans, 2014). Severe adverse events related to the administration of nCRT require in many cases therapy delay, reduction or even termination; moreover, the patient's quality of life may be majorly affected, both acutely and chronically, by impaired organ functions. The literature data related to each gene or pathway investigated in the context of the neo-adjuvant treatment of LARC are reported in the following paragraphs (**Supplementary Table 1**, **Table 1**).

**Table 1:** Main findings from published works on germ-line variants and response to treatment (toxicity and efficacy) in locally advanced rectal cancer (LARC) patients receiving neoadjuvant chemoradiotherapy (nCRT).

Pathway/*Gene*	Polymorphisms	Studies finding an association with the risk of toxicity	Studies that did not find any association with the risk of toxicity	Studies finding an association with the treatment efficacy	Studies that did not find any association with the treatment efficacy
**Fluoropyrimidines Metabolism**					
*DPYD*	DPYD*2A (rs3918290); DPYD*13 (rs55886062); c2846A > T (rs67376798); c.1236G > A/HapB3 rs56038477	Lunenburg et al., 2018			
**DNA Repair**					
*ERCC1*	rs11615			Paez et al., 2011 Sebio et al., 2015;	**Guo et al., 2015** ; **Salnikova and kolobkov, 2016**
	rs3212986			Dreussi et al., 2016a; Sebio et al., 2015; Cecchin et al., 2011	
*ERCC2*	rs13181	Duldulao et al., 2013a	Smith et al., 2017		**Guo et al., 2015** ; **Salnikova and kolobkov, 2016**
*XPA*	rs3176683			Boige et al., 2019;	
*XRCC1*	rs25487	Duldulao et al., 2013a; Osti et al., 2017; Smith et al., 2017		Balboa et al., 2010; Grimminger et al., 2010; Paez et al., 2011; Cecchin et al., 2011; Lamas et al., 2012	Nicosia et al., 2018; **Guo et al., 2015**; **Salnikova and kolobkov, 2016**
*XRCC3*	rs1799794	Osti et al., 2017		Cecchin et al., 2011;	**Guo et al., 2015**; **Salnikova and kolobkov, 2016**
*hOGG1*	rs1052133			Cecchin et al., 2011	
*RAD51*	rs1801320	Osti et al., 2017		Gasinska et al., 2019	
**Folate pathway/Fluoropyrimidines pharmacological target**					
*TYMS*	rs34743033 and rs2853542 (TSER*2 and *3)			Villafranca et al., 2001; Spindler et al., 2007; Balboa et al., 2010; Paez et al., 2010; Hur et al., 2011; Tan et al., 2011; Paez et al., 2011; Lamas et al., 2012; **Salnikova and kolobkov, 2016** **Yang et al., 2017**	Stoehlmacher et al., 2008; Arrazubi et al., 2013; Ulrich et al., 2014;
	rs16430			Arrazubi et al., 2013; Stoehlmacher et al., 2008	Paez et al., 2010; **Yang et al., 2017**
*MTHFR*	rs1801131			Terrazzino et al., 2006;	
	rs1801133	Thomas et al., 2011 (in combination with rs1801131)		Terrazzino et al., 2006; Garcia-Aguilar et al., 2011; Cecchin et al., 2011; Nikas et al., 2015; Dreussi et al., 2016a; **Zhao et al., 2015**; **Salnikova and Kolobkov, 2016**	Ulrich et al., 2014
**Oxidative Stress Detoxification**					
*GSTP1*	rs1695		Osti et al., 2017	Nicosia et al., 2018; Gordon et al., 2006	
**Cellular proliferation/Epidermal Growth Factor**					
*EGFR*	rs712830			Spindler et al., 2006; Spindler et al., 2007 (in combination with rs4444903)	
	rs2227983 rs45608036			**Zhang et al., 2005**; **Zhao et al., 2015**; **Salnikova and Kolobkov, 2016**	
*KRAS*	rs61764370			Sclafani et al., 2015	
*CCDN1*	rs9344			Ho-Pun-Cheung et al., 2007	
*AREG*	rs11942466			Sebio et al., 2015	
**Microenvironment-Related/Inflammation**					
*IL8*	rs4073			Gordon et al., 2006	
*IL13*	rs1800925			Ho-Pun-Cheung et al., 2011;	Xiao et al., 2016
*IL17F*	rs641701			Cecchin et al., 2020	
*NFKB1*	rs28362491	Dzhugashvili et al., 2014		Dzhugashvili et al., 2014	
*TGFB1*	rs1800471	Smith et al., 2017; Schirmer et al., 2012			
	rs1800470			Dreussi et al., 2016a; Gordon et al., 2006	
*PAI1*	rs1050955	Zhang et al., 2015b			
	rs2227631	Zhang et al., 2015b			
*PAR1*	rs32934	Zhang et al., 2015b			
*TNFA*	rs1799964	Zhang et al., 2015a			

In bold are listed meta-analysis and in underlined contrasting results.

### Dihydropyrimidine Dehydrogenase

Dihydropyrimidine dehydrogenase (DPD, *DYPD*) is the enzyme responsible for the *in vivo* 80% detoxification of administered 5-FU, playing a major role as the rate-limiting step in FP metabolism. It has also been reported as the most important player in developing FP-related side effects in patients showing a deficient DPD activity and consequently more prone to the risk of toxicity. The prevalence of DPD partial deficiency in Caucasians is approximately 3%-5% (Etienne et al., 1994). A genetic background related to this inter-individual variability and its role in predicting toxicity occurrence have been described (Van Kuilenburg et al., 1999). Subjects who carry specific genetic variants related to a partial or complete DPD activity loss, may not be able to efficiently detoxify FP at normal rates, and are at greater risk of potentially life-threatening toxicity from standard doses.

The *DPYD* gene is highly polymorphic with 1,732 different genetic variants currently reported in GnomAD (https://gnomad.broadinstitute.org/), and 627 of those being missense variants impacting on the DPD protein sequence. Several studies investigating the role of specific *DPYD* polymorphisms on the risk of FP-related toxicity have been conducted in the past years. A number of them were conducted on large randomized clinical trials (Deenen et al., 2011; Lee et al., 2014; Boige et al., 2016) producing solid evidence on the predictive role of DPYD*2A, DPYD*13, *DPYD*-2846, and *DPYD*-HapB3 on treatment safety. The results were also confirmed in some large meta-analyses (Rosmarin et al., 2014; Meulendijks et al., 2015) and prospective pharmacogenetic guidelines were published providing guidance for dose adjustments based on *DPYD* genotype (Caudle et al., 2017). A first prospective study demonstrated in 2016 that a front-line FP dose adaptation based on the pre-emptive genotyping of DPYD*2A, was effective in preventing toxicity (Deenen et al., 2016).

A gene activity score was proposed for translating *DPYD* genotype into a protein phenotype, considering the diplotype allelic combination of the *DPYD* variants according to a four polymorphisms panel (*DPYD* rs3918290*, DPYD* rs55886062*, DPYD* rs67376798, and *DPYD* rs56038477) (Henricks et al., 2015). A gene activity score was developed to provide a global DPD metabolizing status for each patient and to draw personalized dosing guidelines based on the patients' genotype for the four variants. Currently, the most recent version of shared pharmacogenetic guidelines is based on the definition of the patient gene activity score (Amstutz et al., 2018; Lunenburg et al., 2020). In late 2018 Henricks et al., published the results of a large prospective safety analysis providing strong evidences that the implementation of four *DPYD* polymorphisms genotyping is feasible in clinical practice and that genotype-guided individualized dosing improved patient safety of FP treatment (Henricks et al., 2018). Eventually, this study gave the final input for revising published pharmacogenetic guidelines (suggesting a 50% dose reduction for patients carrying any of the four *DPYD* variants at the heterozygous status) and for recommending *DPYD* genotyping among the strategies for a safer use of FP (https://cpicpgx.org/guidelines/guideline-for-fluoropyrimidines-and-dpyd/; November 2018 Update). Recently, testing of patients for DPD deficiency before starting treatment has been recommended by the European Medicines Agency's safety committee (Pharmacovigilance Risk Assessment Committee) either by measuring the level of endogenous uracil in the blood, or by checking for specific variations in *DPYD* associated with an increased risk of severe side effects (https://www.ema.europa.eu/en/documents/referral/fluorouracil-fluorouracil-related-substances-article-31-referral-ema-recommendations-dpd-testing_en.pdf).

Despite the large amount of published data on the effect of *DPYD* genetic variants on the risk of toxicity both in patients receiving FP monotherapy or different combination chemotherapies (Toffoli et al., 2015; Ruzzo et al., 2017; Dalle Fratte et al., 2018), no specific evaluation of the guidelines application was conducted in patients receiving a concomitant treatment with radiotherapy, as in patients with LARC receiving chemoradiation treatment. FP are commonly administered as short course treatment with lower dosage in the context of chemoradiation treatments, raising perplexities on the opportunity to apply the same genotype-related dose reductions that are prescribed by current guidelines. We demonstrated that the carriers of *DPYD* rs3918290*, DPYD* rs55886062*, DPYD* rs67376798, or *DPYD* rs56038477 variants are still at higher risk of toxicity when treated with chemoradiotherapy. The significant correlation between *DPYD* genotype and toxicity risk was investigated in 828 patients (including 93 patients with LARC) receiving a FP-based chemotherapy in combination with radiotherapy. This study suggests that FP dose reductions should also be applied in *DPYD* variant allele carriers who will start nCRT to prevent severe FP-induced toxicity (Lunenburg et al., 2020).

Among all the pharmacogenomic markers currently available for treatment personalization of patients with LARC, *DPYD* test for FP dosage adaptation is the only validated and recommended. However, clinical implementation of the *DPYD* test prior to FP treatment is not of common practice in most of the health care systems so far. One of the major concerns preventing the translation of *DPYD* pharmacogenetic guidelines in the clinical practice is the lack of formal health technology assessment studies, including cost-effectiveness and cost-consequences evaluation. Our group previously reported that the costs required to manage FP-related toxicity are associated to the patient's *DPYD* genotype. We demonstrated that the mean toxicity management cost per patient is related to the patients *DPYD* gene activity score (based on the four *DPYD* variants) (Fragoulakis et al., 2019; Toffoli et al., 2019). More recently a large prospective trial, provided evidence of the cost-effectiveness of an upfront *DPYD*-guided dose individualization (Henricks et al., 2019).

Probably the ultimate evidence needed to support the introduction of *DPYD* testing and, more generally, of pharmacogenomics in the clinical practice will derive from the ongoing implementation projects (Krebs and Milani, 2019), one of which in Europe (Ubiquitous Pharmacogenomics, U-PGx). U-PGx (www.upgx.eu) is led by a European Consortium of pharmacogenomics experts formed in 2016 with the aim of assessing the clinical utility of implementing a panel of pharmacogenomic markers into routine care. A prospective, block‐randomized, controlled clinical study [PREemptive Pharmacogenomic testing for prevention of Adverse drug REactions (PREPARE)] was funded by the European Commission's Horizon‐2020 program. In such study, a panel of clinically relevant pharmacogenomic markers will be preemptively genotyped and implemented in healthcare institutions across seven European countries and patients' outcome investigated (Cecchin et al., 2017; Van Der Wouden et al., 2017).

The study of rare genetic variants in drug-related “pharmacogenes” represents a promising innovative approach capable to explain and justify the observed variation in overall drug response, not currently explained by common genetic polymorphisms. Furthermore, exome sequencing can accelerate pharmacogenetic discovery by assessing both common (i.e. minor allele frequency, MAF >5%) and rare (MAF < 1%) mutations in virtually all genes in an individual at relatively low cost (Gordon et al., 2014; Ingelman-Sundberg et al., 2018). In the future, additional novel and rare variants significantly impacting the DPD activity could be integrated into available pharmacogenomic algorithms to further improve the safety of FP administration.

### DNA Repair Pathway

**Figure 2:**
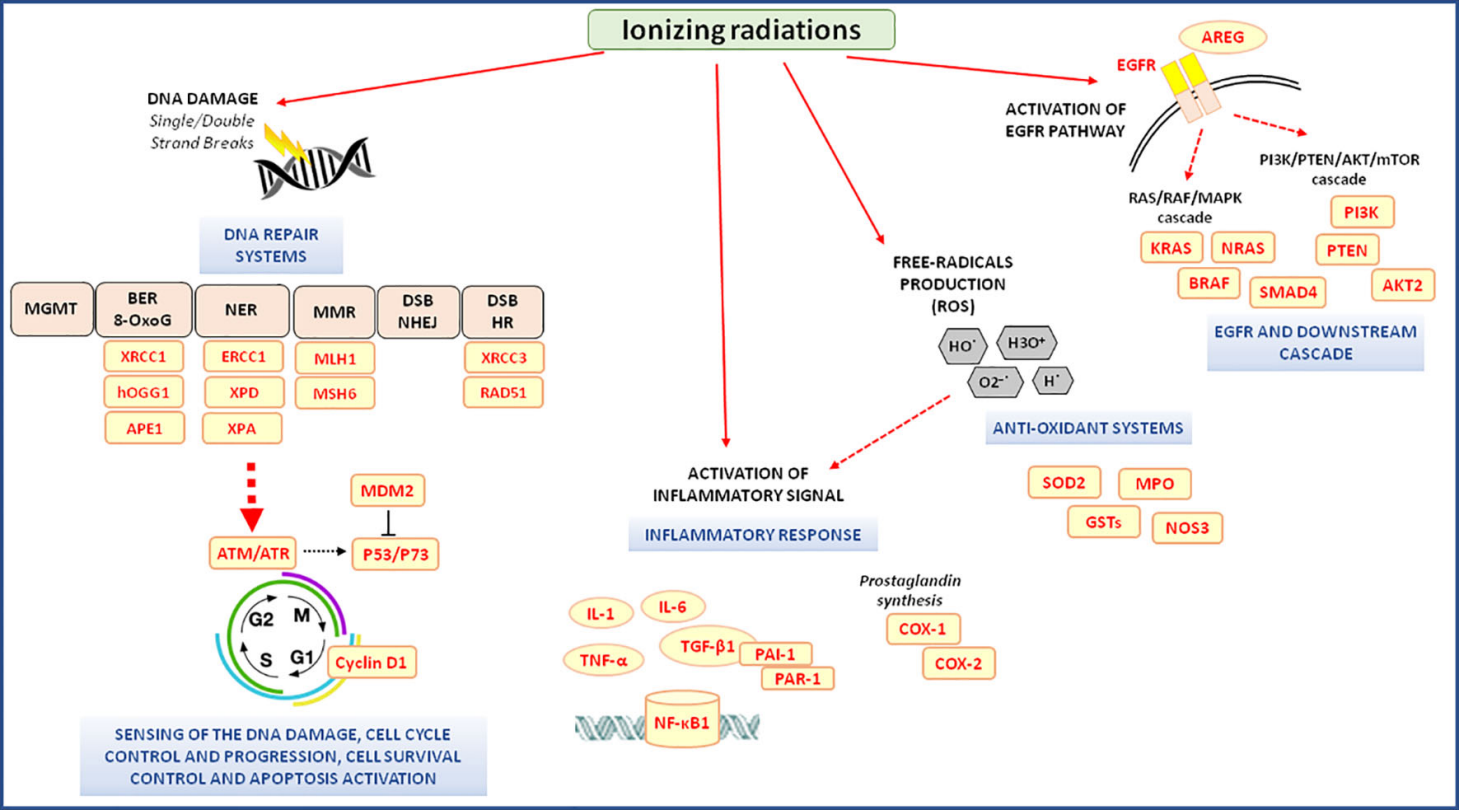
Radiotherapy-related molecular pathways associated with the clinical outcome of neo-adjuvant chemoradiotherapy in locally advanced rectal cancer patients. 8-oxoG, 8-oxoguanine; AKT, v-akt murine thymoma viral oncogene homolog; APE1, apurinic/apyrimidinic endodeoxyribonuclease 1; AREG, amphiregulin; ATM, ATM serine/threonine kinases; ATR, ATR serine/threonine kinases; BER, base excision repair; COX-1, -2; cyclooxygenase 1, -2; DSB, double-strand breaks; EGFR, epidermal growth factor receptor; ERCC1, ERCC excision repair 1; GSTs, glutathione S-transferases; hOGG1, 8-oxoguanine DNA glycosylase; HR, homologous recombination; MDM2, MDM2 proto-oncogene; MGMT, O6-methylguanine DNA methyltransferase; MLH1, mutL homolog 1; MMR, DNA mismatch repair; MPO, myeloperoxidase; MSH6, mutS homolog 6; mTOR, mammalian target of rapamycin; NER, nucleotide excision repair; NF-κB1, nuclear Factor Kappa B Subunit 1; NHEJ, non-homologous end joining; NOS3, nitric oxide synthase 3; P53, tumor protein p53; P73, tumor protein p73; PAI-1, plasminogen activator inhibitor type 1; PAR-1, protease-activated receptor-1; PI3K, phosphoinositide-3-kinase; PTEN, phosphatase and tensin homolog; RAD51, RAD51 recombinase; ROS, reactive oxygen species; SMAD-4, SMAD family member 4; SOD2, superoxide dismutase 2; TGF-β1, transforming growth factor beta 1; TNF-α, tumor necrosis factor-alpha; TRBP, trans-activation-responsive RNA-binding protein; XPA, xeroderma pigmentoso complementation group A; XPD, xeroderma pigmentoso complementation group D; XRCC1, -3, x-ray repair cross-complementing 1, -3.

RT exerts its cytotoxic action mainly by acting on DNA. If the DNA damage after irradiation is not completely repaired, cell death will occur either by apoptosis, mitotic catastrophe, or senescence (Maier et al., 2016) (**Figure 2**). At least five DNA repair molecular systems, each operating on a specific type of DNA damage, are involved in the repair of radiation-induced damage: direct reversal as the O6-methylguanine DNA methyltransferase–base repair; base excision repair (BER) including the repair of modifications generated by reactive oxygen as the 8-oxoguanine; nucleotide excision repair (NER); DNA mismatch repair; and double-strand breaks (DSB) repair that involved two main pathways, the non-homologous end joining (NHEJ) and homologous recombination (HR). A genetically determined defect in the capacity to repair RT induced damage has been reported (Lavin, 1998; Mckinnon and Caldecott, 2007; Mizutani and Takagi, 2013).

Three meta-analyses reported the role of DNA repair genetic variants as predictive markers of the risk to develop severe radiation-induced toxicity, although including patients withunselected solid cancers and not specifically LARC. including patients with unselected solid cancers and not specifically LARC. Particularly these meta-analyses demonstrated a significant involvement of polymorphisms in the ERCC excision repair 2 [*ERCC2*, also known as xeroderma pigmentosum group D (XPD), i.e., rs13181, Lys751Gln], x-ray repair cross-complementing 1 (*XRCC1*, i.e., rs25487, Arg399Gln), x-ray repair cross-complementing 3 (*XRCC3*, i.e., rs861539, Thr241Met; rs1799794, located in the 5'UTR) and ATM serine/threonine kinases (*ATM*, i.e., rs1801516, Asp1853Asn) genes in modulating the risk of radiotoxicity (Dong et al., 2015; Song et al., 2015a; Song et al., 2015b). However, the results of the meta-analyses are not fully transferable to the specific context of the radiation neo-adjuvant therapy of patients with LARC, where most promising data regard the *XRCC1* rs25487 variant. A concordant association between the presence of a polymorphic rs25487-A (Gln) allele and an increased risk to develop grade ≥3 toxicity, and skin toxicity of any grade was reported by three different research papers (Duldulao et al., 2013a; Osti et al., 2017; Smith et al., 2017). XRCC1 is one of the most relevant members of the BER pathway and its missense variation rs25487 was associated with a decreased DNA repair capacity, supporting an impact on the risk of toxicity (Duldulao et al., 2013a).

Genetic polymorphisms in *RAD51* recombinase (*RAD51*, i.e., rs1801320) and *XRCC3* (i.e. rs1799794), two members of the DSB-HR repair pathway, were also associated to toxicity risk. The polymorphic *RAD51* rs1801320-C allele resulted predictive of an increased risk of grade ≥3 abdominal/pelvis pain toxicity and acute skin toxicity of any grade, while the *XRCC3* rs1799794-G allele was associated with increased risk of grade ≥3 urinary frequency/urgency, acute skin toxicity of any grade and higher rates of fatigue (Osti et al., 2017). The *RAD51* rs1801320 G to C substitution in the untranslated (UTR) 5' region of the gene was shown to affect the mRNA stability leading to a lower protein expression and less effective DSBs repair (Gasinska et al., 2019). A genetically impaired *RAD51* expression has been reported to impact the individual radiosensitivity, and the rs1801320 variant was correlated with the risk of radiation-induced toxicity also in other tumor settings (Osti et al., 2017).

The missense variation rs13181 (Lys751Gln) in *ERCC2*, a member of NER system, was also investigated. The *ERCC2* rs13181-AA (Lys/Lys) genotype, resulted associated with an increased rate of grade ≥3 toxicity after a radiotherapy including treatment (Duldulao et al., 2013a). A following study aiming at validating the predictive role of *ERCC2*-rs13181 on radiotoxicity risk in patients with LARC (Smith et al., 2017) did not confirm those results.

### Inflammation Pathway

In the context of chemoradiation treatment, inflammation-related genes have been considered as a target for the pharmacogenomic research, though the clinical setting of LARC has been poorly investigated up to date (**Figure 2**). The most promising results are related to the transforming growth factor beta 1 (TGF-β1), a cytokine that initiates and promotes acute and late radiation-induced side effects and is thus considered a major player in the inflammation process triggering. Exposure to radiation activates TGF-β1 *via* the generation of reactive oxygen species, and a sustained over-expression of TGF-β1 has been found in irradiated tissue. It was also investigated the role of rs1800471 in TGF-β1. The study of Schirmer et al., (2012), including two independent cohorts (discovery and replication) of Caucasian patients with LARC receiving neo-adjuvant radiation combined with 5-FU-based chemotherapy, evaluated the effect of *TGFB1* rs1800471 on the acute organ toxicity (quality of life-impairing acute organ toxicity, QAOT). In both cohorts, *TGFB1* rs1800471 resulted a significant predictor of QAOT, with all the patients carrying the polymorphic TGFB1 rs1800471-C (Pro25) allele experiencing QAOT. These data were confirmed by a subsequent study (Smith et al., 2017), performed in a similar cohort of Caucasian patients with LARC treated with FP-based CRT. The study reported that *TGFB1* rs1800471-C (Pro25) allele was associated with an increased risk of severe (grade ≥3) toxicity. The functional effect of the *TGFB1* rs1800471 polymorphism on the encoded protein has not been fully clarified yet. The polymorphism is in a genetic region encoding for the signaling portion of the peptide involved in the transport of the protein across the membrane of the endoplasmic reticulum. Preliminary *in silico* analysis has suggested that the presence of a proline at position 25, caused by rs1800471-C allele could affect the cleavage of the peptide, increasing TGFβ1 secretion and signaling (Schirmer et al., 2012). Another study including Chinese patients with LARC receiving nCRT demonstrated an effect on the toxicity risk of some polymorphisms in genes encoding for plasminogen activator (PA) inhibitor type 1 (PAI-1) and protease-activated receptor-1 (PAR-1). These two proteins, whose expression is activated by the TGFβ1-related pathway, contribute to acute and late radiation-induced injury (Zhang et al., 2015b). By multivariate analysis, *PAI-1* rs1050955-GG and *PAR-1* rs32934-CT genotypes were associated with lower risk of grade ≥2 diarrhea, fecal incontinence (i.e., anal toxicity) and other toxicities, while the *PAI-1* rs2227631-GG was associated with increased risk of grade ≥2 incontinence (Zhang et al., 2015b). Those polymorphisms are placed in the genes regulatory regions and may affect gene expression in different ways. The 3'UTR *PAI-1* rs1050955 variant is located at a microRNA binding site, whereas *PAI-1* rs2227631 and *PAR-1* rs32934 are located in the 5′-flanking region of the gene and may change the binding site of specific transcriptional factor (Zhang et al., 2015b). These data support a major causative role of TGFβ1 and its downstream mediators (i.e., PAI-1, PAR-1) on the risk of radiation–related toxicity, and a better elucidation of the predictive role of the related polymorphisms is warranted. However, the specific role of the genetic polymorphisms in the pathway mediators *PAI-1, PAR-1* is proved by a single study and the lack of independent validation results prevents to draw final conclusion.

A second cytokine of interest is the nuclear factor κB1 (NFKB1), a transcriptional factor that plays a key role in the activation of the inflammatory response and regulates various biological defense processes, including innate and adaptive immune responses, acute phase reaction and apoptosis (Karban et al., 2004). The *NFKB1* rs28362491 variant, consisting in an insertion/deletion of four bases in the promoter region of the gene, seems to decrease *NFKB1* transcription level by reducing the binding capacity of some transcription factors to the gene promoter (Karban et al., 2004; Riemann et al., 2007). Regarding the predictive role of *NFKB1* rs28362491 marker, literature provides only one study. The polymorphism was associated to the risk of radiation-induced toxicity. Particularly, the *NFKB1* rs28362491-DEL/DEL genotype was associated with an increased risk of grade ≥2 dermatitis and proctitis. The rs28362491-DEL containing haplotype also showed a correlation with an increased risk to develop clinically significant grade≥2 acute organ toxicity (Dzhugashvili et al., 2014).

The pro-inflammatory cytokine tumor necrosis factor-alpha (TNF-α, encoded by *TNFA*) is another key molecule involved in the mediation of the inflammatory signal activated by ionizing radiation. As far as the predictive role of *TNFA* polymorphism in LARC is concerned, only one hypothesis generating study is currently available. The T allele of the *TNFA* rs1799964, located in the 5′-flanking region of the gene, was associated with increased TNF-α mRNA expression *in vitro*. It was reported to be associated with an increased risk of total grade ≥2 acute toxicity after chemoradiotherapy in Chinese patients with LARC (Zhang et al., 2015a).

Despite the promising aforementioned data about the potential involvement of some polymorphisms in genes implicated in the inflammation process in mediating the risk of toxicity in patients with LARC treated with nCRT, it must be underlined that most of the results are only reported in single exploratory studies, as for the case of polymorphisms in *PAI-1, PAR-1*, *NFKB1, TNFA*. Therefore, they should be further tested in independent validation studies in order to be then considered as effective biomarkers for a clinical translation.

## Neo-Adjuvant Radio-Chemotherapy Combination Treatment in LARC and Pharmacogenomic Markers of Efficacy

The most compelling clinical need in patients with LARC is represented by a better understanding of the inter-individual unpredictable heterogeneity in the tumor response to nCRT. The prospective identification of patients who have a higher likelihood of responding to nCRT could be important for decreasing treatment morbidity and improving survival and local disease control. On the other hand, alternative intensified therapeutic approaches could be offered to patients who are unlikely to respond. Both germline and somatic genetic variants could play a role in defining the individual chance to get an effective response to treatment or the risk of disease recurrence (**Supplementary Table 2**, **Table 1**). A revision of the available literature evidence on genetic polymorphisms and somatic variants that could be considered to predict tumor response to treatment in LARC is reported below.

### Thymidylate Synthase

Thymidylate synthase (TS, *TYMS*), an enzyme with a critical role in the DNA synthesis process, is the primary target of FP and its expression level was suggested to inversely correlate with the tumor sensitivity to FP (Johnston et al., 1995; Libra et al., 2004). Some common polymorphisms with an impact on the protein expression level and, in turn, with a hypothesized effect on an FP-based treatment outcome, were described in *TYMS*.

The most studied *TYMS* polymorphism consists of regulatory tandemly repeated sequences (VNTR) of 28–base pairs (rs34743033) in the 5' untranslated region of the gene and acts as an enhancer to the TYMS promoter (TYMS enhancer region[TSER]). TYMS expression is affected by the number of tandem repeats that are directly correlated with the enzyme expression level. Alleles with two or three repeats are the most common (TSER*2 or *2R* and TSER*3 or *3R)*. The second repeat of the 3R allele hosts a functional G > C single-nucleotide polymorphism (rs2853542). The 3R(G) allele has been correlated with a more efficient transcription activity while the transcription efficiency of the 3R(C) allele is the same as that of the 2R allele. TSER and rs2853542 are often combined to identify two groups of alleles: *TYMS* high-expression allele (2R/3RG, 3R/3RG and 3RG/3RG) and low-expression allele (2R/2R, 2R/3RC and 3RC/3RC) (Thomas et al., 2011). A third polymorphism that has been studied for its effect on the protein expression is a 6 bp insertion at the nucleotide 1494 in the 3′UTR of the gene. It was reported to be associated with an increased *TYMS* tumor expression that may decrease the chemosensitivity to FP (1494del6, rs16430, or rs34489327).

In the last 10 years, the association of *TYMS* polymorphisms with the tumor response to nCRT in LARC has been under investigation in literature. Although several studies showed an inconsistency of statistically significant results, in general *TYMS* high-expression alleles were associated with a worse response to nCRT and unfavorable prognosis. On the other side, *TYMS* low-expression alleles have been overall associated with better response and prognosis.

According to an abstract presented in 2001 from Villafranca et al., (2001) TSER*3 homozygotes had a lower probability of downstaging compared with TSER*2 homozygotes or *2/*3 heterozygotes (22% vs 60%) and a trend toward improved 3-year DFS was also detected in the *2/*2 and *2/*3 groups, compared with that in the *3/*3 group (81% vs 41%). The group of Spindler in 2007 demonstrated that 53% of *2/*2 patients experienced a 53% p-CR compared with 26% of *2/*3 and only 17% of patients with the TSER*3/*3 variants (Spindler et al., 2007). The contribute of the 28-bp VNTR in the *TYMS* 5'UTR was considered along with the G > C SNP by Hur et al., reporting a significant difference in tumor response between low and high-expressing groups in 44 South Korean patients with LARC. Thirteen out of 14 patients in the low-expression genotype group exhibited a significantly greater tumor downstaging rate, as compared with 12 of 30 patients in the high-expression group (Hur et al., 2011). Based on such consistent results a prospective tandem phase II study was designed in 2011 by Tan and colleagues. *TYMS* genotyping was used to guide nCRT where carriers of three or more repeats in the *TYMS* 5'UTR VNTR were treated with a chemotherapy intensification program (Tan et al., 2011). TSER*2/*2 or TSER*2/*3, were treated with the standard of care hypothesizing a favorable response to 5-FU, while TSER*3/*3 or TSER*3/*4 genotypes who were unlikely to derive significant benefit from a FP-based CT were treated with irinotecan in addition to standard 5-FU/CRT. This genotype-based strategy appeared to be successful, increasing treatment efficacy in poor-responder patients. Downstaging and complete tumor response rates reached 64.4% and 20% for standard of care and 64.5% and 42% for irinotecan-treated patients, respectively. However, this increase in efficacy came at the cost of higher rates of grade 3 to 4 toxicities, delay rates and hospitalization (Tan et al., 2011).

Only a few studies deriving from one research group reported contrasting results, highlighting an association between *3/*3 genotype and a higher response rate. The first study identified the *3/*3 genotype as an independent prognostic factor for improved survival in 2010 (Paez et al., 2010). A successive study conducted in 2011 on a larger group of patients reported as well a higher response rate (pathologic complete remission and microfoci residual tumor) for *TYMS* *3/*3 genotype compared to *2/*2 or *2/*3 in the amount of 59% vs 35%, and longer median progression-free survival (PFS) and OS (Paez et al., 2011). A significantly improved pathologic response among carriers of *TYMS* high-expressing alleles as compared with low-expressing alleles was also found by Lamas et al., (2012).

Regardless, two systematic reviews and metanalyses were published in 2016 (Salnikova and Kolobkov, 2016) and 2017 (Yang et al., 2017) assessing that *TYMS* *2/*3 genotype is associated with the response to nCRT in rectal cancer and that patients with a *2/*2 or *2/*3 genotype might benefit more from nCRT than others. The initial evidence of results heterogeneity for *TYMS* rs34743033 resolved after the exclusion of the data of Paez et al., (Paez et al., 2011; Salnikova and Kolobkov, 2016). However, the authors suggest that such results are not conclusive due to discrepancies among the studies analyzed in the nCRT administration and surgery strategies, sample size and ethnicities. A significant bias could also have derived from the source tissue (either tumor or normal) used for germinal DNA extraction.

Eventually, 3'-UTR 6-bp rs16430 was significantly associated with DFS by Arrazubi et al., (2013), while only a trend towards tumor response in patients receiving 5-FU-based nCRT, was described by Stoehlmacher et al., (2008). The metanalysis from Yang et al., (2017) did not found 1494del6 to be associated with the response to nCRT.

### Methylenetetrahydrofolate Reductase

Methylenetetrahydrofolate reductase (encoded by *MTHFR* gene) is a fundamental enzyme in the folate cycle, important for DNA synthesis and repair (Toffoli et al., 2003) and hypothesized to be another important target of the FP pharmacological activity. MTHFR catalyzes the reduction of 5,10-methylenetetrahydrofolate to 5-methyltetrahydrofolate, the methyl donor for methionine synthesis from homocysteine. Two common non-synonymous variants in *MTHFR*, rs1801133 (677C > T, Ala222Val) and rs1801131 (1298A > C, Glu429Ala), alone or in combination, were reported to affect the activity of the enzyme and hence the folate distribution (De Mattia and Toffoli, 2009; De Re et al., 2010). *MTHFR* rs1801133-CC wild genotype was associated with a major response to nCRT compared with *MTHFR* rs1801133-TT genotype (Salnikova and Kolobkov, 2016). The radiotherapy effect was also hypothesized to be affected by the action of MTHFR. Specifically, the reduced MTHFR activity, deriving from the aforementioned polymorphisms, could lead to an enhanced availability of non-methylated folate substrates for *de novo* synthesis of nucleotides, which could preserve DNA integrity from ionizing radiation, reducing genetic instability and thus preserving its efficacy. A number of studies were published with pretty consistent results about the detrimental role of *MTHFR* rs1801133 on the outcome of a CRT treatment. In particular, the *MTHFR* rs1801133-CC genotype was demonstrated to be more sensitive to the therapy, whereas the effect of *MTHFR* rs1801131 is still controversial.

In two studies on patients with LARC, *MTHFR* rs1801133-T allele was significantly associated with a lower chance to get a TRG ≤ 2 (Cecchin et al., 2011) and with non-pCR when in homozygosity (Garcia-Aguilar et al., 2011). In 2014 Urlich et al., documented a non-significant trend toward worse DFS and OS for *MTHFR* rs1801133-TT compared to patients with CC, although not supported by a significant association with OS or DFS. Such trend became significant for both endpoints in patients receiving 5-FU *via* protracted venous infusion 42 days before and 56 days after radiotherapy (Ulrich et al., 2014). Nikas et al., in 2015 reported that patients with *MTHFR* rs1801133-CC were 2.91 times more likely to benefit from nCRT, and 3.25 times more likely not to experience recurrence of the disease compared to heterozygous (CT) or homozygous (TT) genotypes (Nikas et al., 2015). The study from Terrazzino et al., identified the haplotype *MTHFR* rs1801133-T/rs1801131-A as an independent predictor of tumor regression at univariate and later confirmed at multivariate analysis. Patients not carrying the *MTHFR* rs1801133-T/rs1801131-A haplotype displayed a higher response rate (Terrazzino et al., 2006). Eventually the systematic review and metanalysis from Zhao et al., draw the line suggesting that *MTHFR* rs1801133 might be correlated with the tumor response under the recessive model (CC vs. CT/TT) in overall analysis, rectal cancer, and TRG1–2 vs. 3–5 group. *MTHFR* rs1801131 showed no significant association with the tumor response to nCRT (Zhao et al., 2015).

It has also been hypothesized that the effect of *MTHFR* SNPs could change according to specific combination drug therapies given with radiotherapy. A study by Dreussi et al., (2016a) compared the effect of a set of SNPs, including *MTHFR* rs1801133, in patients with LARC receiving an FP-based nCRT with or without oxaliplatin. They demonstrated a differential effect of the polymorphisms on the tumor response in the two subgroups, thus suggesting an effect of drug-drug interactions on the pharmacogenomic of LARC treatment.

*MTHFR* polymorphisms were also anecdotally associated with FP-related toxicity. A study published in 2011 by Thomas et al., investigated the effect of *MTHFR* rs1801133 and rs1801131 genetic markers with the outcome of a chemoradiation treatment. *MTHFR* haplotypes *(*rs1801133-*C/*rs1801131-C*)* and diplotypes (CA–TA and TA–TA) showed a protective or a detrimental effect on the incidence of severe diarrhea or mucositis, respectively (Thomas et al., 2011).

### DNA Repair Pathway

The major role of germline variation in the DNA repair pathway on the response to radiotherapy has been already mentioned above for its association with a differential risk to develop adverse side effects after a chemoradiation treatment. The same genetic polymorphisms were investigated for their potential role in identifying good or poor responders to radiotherapy-based treatments. However, the results obtained up to date are far from their final conclusions. The missense *XRCC1*-rs25487 variant was investigated in a number of studies that generated heterogeneous results (Balboa et al., 2010; Grimminger et al., 2010; Cecchin et al., 2011; Paez et al., 2011; Lamas et al., 2012). The heterozygous rs25487-AG genotype was reported to be associated with both a higher (Grimminger et al., 2010) and a lower (Lamas et al., 2012) response rate. The *XRCC1* rs25487-AA genotype was associated either with an improved tumor response (Balboa et al., 2010) or with a decreased PFS by other authors (Paez et al., 2011). Interestingly, the work of Balboa et al., (2010) raised the problem of the source of genomic DNA used in the different studies for *XRCC1* genotyping (i.e. blood or tumor tissue). In their investigation, the authors demonstrated a discrepancy between the *XRCC1* rs25487-AA genotype as determined in healthy or tumor cells. The authors found an elevated rate of tumor specific genomic alterations (i.e., a loss of heterozygosity, gain of allele), in the chromosomal region containing DNA repair encoding genes (i.e., *XRCC1, ERCC1, ERCC2*), with the *XRCC1* locus presenting the highest percentage of aberrations. These findings highlighted a potential limitation in interpreting the results of the pharmacogenomic studies currently available.

The gene encoding polymorphisms for *ERCC1* (a NER member) were also analyzed by a number of studies, generating conflicting results. The TT genotype of the synonymous rs11615 (Asn118Asn) polymorphism, impacting the mRNA expression level of *ERCC1*, (Yu et al., 2000), resulted associated with a higher rate of R1-R2 circumferential rectal margin resection, and higher recurrence rate (Paez et al., 2011). Opposite results were produced by another study, where the same *ERCC1* rs11615-TT genotype resulted associated with higher rate of pathological complete response by multivariate analysis (Sebio et al., 2015). *ERCC1* polymorphism (i.e. rs3212986, located in the 3'UTR) was also highlighted for its association with complete tumor response to treatment (Cecchin et al., 2011; Dreussi et al., 2016a) but the results were not concordant with the findings of another published study (Sebio et al., 2015).

The impact of a set of DNA repair genetic markers (i.e., *XRCC1*-rs25487, *XRCC1*-rs179978, *XRCC3*-rs861539, *ERCC1*-rs11615, *ERCC2*-rs13181) on the tumor pathological response was analyzed by a meta-analysis (Guo et al., 2015), including five studies for a total of 265 Caucasian patients with LARC. The meta-analysis found no association between the polymorphisms and the response to radiotherapy in the context of LARC multimodality treatment. However, it should be considered that the small sample size of each single study, together with their significant methodological heterogeneity (genotyping strategy, study design, treatment schedule and dosage, combination with different chemotherapeutics drugs, clinical monitoring), could have affected the possibility to draw a common conclusion.

Additional studies were published exploring the predictive/prognostic effect of polymorphisms in other genes involved in the DNA repair pathways (i.e., *hOGG1*, *APE1*, *RAD51*, and *XPA*). Despite the encouraging results of the investigations on their potential impact on the tumor response phenotype, a final consensus was not achieved, and the clinical value of the markers should be independently validated in further studies.

The variant G allele of the missense rs1052133 (Ser326Cys) polymorphism, impairing the functionality of hOGG1 (Vodicka et al., 2007), was indicated to be a detrimental factor for the pathological tumor response to radiation therapy (Cecchin et al., 2011). The minor G allele of the missense rs1130409 (Asp148Glu) variation in *APE*-1, was instead associated with an increased response rate to radiotherapy at high dose (Dreussi et al., 2016a).

The functionally defective *RAD51* rs1801320-CC genotype was recently reported to be associated with a better outcome after short-course radiotherapy in term of both a longer OS and a lower risk to develop local recurrence and distant metastasis (Gasinska et al., 2019). This evidence supports the hypothesis that a defective repair of the radiation induced damage on DNA, determined by a genetic polymorphism, could increase the treatment efficacy with an improved patients' prognosis. Interestingly, patients with a *RAD51* rs1801320-CC genotype presented also a different tumor tissue phenotype with lower glucose transporter 1 expression, moderate differentiation, lower Ku70 expression, lower aneuploidy, and higher P53 protein expression. Moreover, patients' gender, in association with *RAD51* genotype and Ku70 expression, was shown to have an impact on OS. The findings of this study represent one of the first evidence of an association between a specific genetic background related to DNA repair (i.e., *RAD51* genotype) and a peculiar rectal cancer molecular phenotype and suggest a gender-related difference in the therapy outcome.

Another recent study by Boige et al., (2019) suggested that the *xeroderma pigmentosum group A (XPA)* rs3176683 polymorphism may help identifying patients who could benefit from adding oxaliplatin to a capecitabine-based nCRT due to its predictive effect for tumor response to oxaliplatin-based nCRT. The probability of tumor response was seven times higher in patient with *XPA* rs3176683-TT genotype treated with CAPOX (i.e., capecitabine and oxaliplatin). However, the polymorphism failed to be associated with the patients' prognosis.

### Oxidative Stress/Detoxification Pathway

Since the radiotherapy exerts part of its cytotoxic effect by generating reactive oxygen species (ROS), genetic variations in oxidative stress-related enzymes have been also investigated as potential predictors of tumor response to treatment. However, the studies published up to date in LARC are sporadic and do not allow to draw final conclusion on the actual predictive or prognostic role of the markers investigated in *NOS3, MPO, SOD2*, and *GST*.

The missense rs1799983 (Glu298Asp) polymorphism in the gene encoding endothelial nitric oxide synthase (eNOS, *NOS3*), was related to ROS generation and patients OS. The same study also highlighted an association between the low ROS producing A-allele of the rs2333227 variant, located in the 5'flanking of the *myeloperoxidase (MPO)* gene, and a longer OS (Funke et al., 2009). Another potential predictive marker pointed out by literature is the missense rs4880 (Ala16Val) variant in SOD2 encoding gene. SOD2 plays a central role in the detoxification of ROS and its higher expression was reported to inhibit ROS-induced activation of NF-κB and to potentially increase the sensitivity to ionizing radiations (Ho-Pun-Cheung et al., 2011). In agreement with this hypothesis, the rs4880-T (Val), associated with lower SOD2 activity, was linked to a worse response to radiation therapy (Ho-Pun-Cheung et al., 2011).

Glutathione‐S‐transferases (GSTs) is a multigene family of phase II metabolic enzymes employed in the detoxification of reactive oxygen intermediates produced by a wide variety of potentially toxic and carcinogenic compounds, by conjugation with glutathione (Nicosia et al., 2018). The missense polymorphism 313A > G (Ile105Val, rs1695) in *GSTP1* was proposed, in a study from Nicosia et al., (2018) to be a predictive and prognostic marker. Among a population of 80 patients with LARC, those with a 313AA genotype for *GSTP1* rs1695presented a rate of 26.6% of pCR compared to 8.5% of the 313AG/GG population. Patients harboring at least a 313A allele presented a 5- and 8-year cancer-specific survival longer than those with 313GG genotype (87.7% and 83.3% vs. 44.4% and 44.4%, respectively). Only a trend for an improved OS in the rs1695-A allele carriers was reported.

### Epidermal Growth Factor Receptor

EGFR was demonstrated to have a pivotal role in LARC as well as in the colorectal cancer carcinogenesis (Toffoli et al., 2007; Kim and Eng, 2012). Moreover, EGFR and related pathways, are activated by ionizing radiation, and have been suggested to be a druggable target for the neo-adjuvant treatment of LARC. However, the association of FP with cetuximab in the chemoradiation treatment was not successful. Nonetheless, germline polymorphisms in *EGFR* were investigated for their potential predictive or prognostic effect. The most studied functionally relevant *EGFR* polymorphisms in LARC are the following: 1. rs45608036, a (CA)n repeat in the intron 1 of the gene that alters EGFR expression *in vitro* and *in vivo*; 2. rs2227983, a missense substitution at codon 497 (Arg497Lys) that leads to attenuation in ligand binding, growth stimulation, tyrosine kinase activation, and induction of proto-oncogenes myc, fos, and jun; and 3. rs712830, a single nucleotide change located in the Sp1 binding site of the regulatory *EGFR* promoter region that impact *EGFR* transcription (Zhang et al., 2005; Spindler et al., 2006; De Mattia et al., 2015). The rs712830-GG genotype resulted associated with a poorer response to chemoradiotherapy (Spindler et al., 2006). In the same study, the rs712830-GG genotype was also associated with higher EGFR expression by immunohistochemistry.

In another exploratory study, both the *EGFR* rs2227983-GG (Arg/Arg) genotype and the rs45608036-CA repeats (genotype with both alleles <20 CA repeats) showed a tendency towards an increased risk of local recurrence after radiotherapy. The combination in the same patient of an rs2227983-G (Arg) and an rs45608036 < 20 CA repeats alleles was associated with the highest risk of local recurrence (Zhang et al., 2005). A subsequent meta-analysis, including about 350 Caucasian patients with LARC and evaluating the role of *EGFR* rs2227983 and rs45608036 on the response to chemoradiation therapy, showed a trend for a detrimental effect on the response to treatment of the *EGFR* shorter (S) alleles (Zhao et al., 2015). In another meta-analysis, including more than a thousand Caucasian patients with LARC, a different frequency of distribution of the *EGFR* rs2227983-A allele among responder and non-responder has been detected (Salnikova and Kolobkov, 2016). Further analyses are required to clarify whether specific *EGFR* polymorphisms can have a clinical utility in predicting the outcome of a chemoradiation therapy in LARC.

Within the EGFR-pathway, preliminary results have also been generated for some germline genetic markers in the downstream effector KRAS proto-oncogene (*KRAS*) and the EGFR-ligand amphiregulin (*AREG*). A 3'UTR *KRAS* variant, named LCS6 (rs61764370) was reported to alter the epigenetic transcriptional control of KRAS. The polymorphism was associated with an impaired capacity of the mature let-7 miRNA to bind the KRAS-encoding mRNA, potentially leading to KRAS up-regulation and downstream cellular pathway signaling interference (De Mattia et al., 2015). In patients with LARC, the results of the retrospective EXPERT-C trial evidenced that the minor *KRAS* rs61764370-G allele was associated with a higher rate of complete response after nCRT independently from cetuximab administration (Sclafani et al., 2015). The same polymorphic rs61764370-G-allele showed also an association trend for an improved 5-years PFS and OS. By a subgroup analysis, the favorable prognostic effect of the rs61764370 variant seemed to be limited only to patients with a *KRAS* mutated tumor, opening the possibility of identifying subgroups of good responder patients within that category of patients (*KRAS* mutated) presenting an overall unfavorable prognosis.

Some hypothesis-generating data were also published about the role of genetic polymorphisms in some downstream effectors and ligands cooperating in the EGFR pathway. These data are reported in single studies lacking independent validation and therefore requiring further investigation to assess their real clinical value.

Specifically, the intergenic rs11942466 polymorphisms located in the *AREG* gene region was associated with a higher rate of pCR. Through a classification and regression tree analysis, including some polymorphisms in the EGFR pathway and DNA repair genes, the *AREG* rs11942466 variant represented the most important marker for identifying the complete responder patients (Sebio et al., 2015). The phosphoinositide-3-kinase (PI3K)/phosphatase and tensin homolog (PTEN)/v-akt murine thymoma viral oncogene homolog (AKT)/mammalian target of rapamycin (mTOR) cascade is another downstream signaling pathway potentially activated by EGFR. The role of genetic polymorphisms in five genes belonging to the PI3K/PTEN/AKT/mTOR pathway [i.e, *PI3K catalytic subunit alpha (PIK3CA), PTEN, AKT1, AKT2, FRAP1* encoding for mTOR] on the response to nCRT in LARC was recently investigated for the first time. The *PTEN* rs12569998-G allele was associated with an increased tumor response rate, while the *AKT2* rs8100018-C allele with a decreased recurrence risk and a higher 5-years DFS rate (Peng et al., 2018). *PTEN* plays a crucial role in the DNA damage repair and in the cellular response to DNA damage, whereas *AKT2* modulates the cell survival signals, important mechanisms for the response to nCRT in LARC.

Additional genetic markers affecting the cell cycle control, apoptosis and proliferation rate were evaluated in single exploratory studies. The rs9344 polymorphism in cyclin D1 (*CCND1)*, controlling the G_1_/S checkpoint of the cell cycle, is located at codon 242 in the exon-4/intron boundary of *CCND1* gene and is responsible for alternate splicing of transcripts with different half-lives (Bitterman et al., 2015). The *CCDN1* rs9344 marker was found to be an independent predictor of response to neo-adjuvant radiotherapy, with the A-allele being associated with an increased response rate and a lower risk of local failure (Ho-Pun-Cheung et al., 2007). Moreover, the combination of *CCDN1* rs9344 with specific clinical-pathological parameters (i.e., post-therapeutic lymph node status) generated a prognostic index that accurately distinguished subgroups of patients with different recurrence-free survival and OS. Other data suggested that the p73 G4C14 → A4T14 marker could be an additional factor influencing the outcome of nCRT in LARC. G4C14 → A4T14 is a dinucleotide polymorphism consisting of two linked variants (rs2273953 and rs1801173, respectively) located at position 4 (G to A) and 14 (C to T) in the 5′-UTR of exon 2, just upstream the initial start codon of the *tumor protein p73* (*TP73*, encoding P73) gene. The G4C14 → A4T14 polymorphism was supposed to impact the gene expression of p73, implicated in the regulation of the balance between pro- and anti-apoptotic signals (Loof et al., 2009). The GC/GC genotype was showed to be associated with a lower expression of p53 and Survivin, another regulator of the apoptosis and cell-cycle, and to be tendentially related to a longer DFS. The combination of a GC/GC genotype with a negative p53 expression and a weak expression of Survivin, predicted a longer DFS as compared to other genotype/phenotype combinations (Loof et al., 2009). All of these markers could be considered of interest, but only future validation work will eventually assess their clinical value.

### Microenvironment-Related Pathways

**Figure 3:**
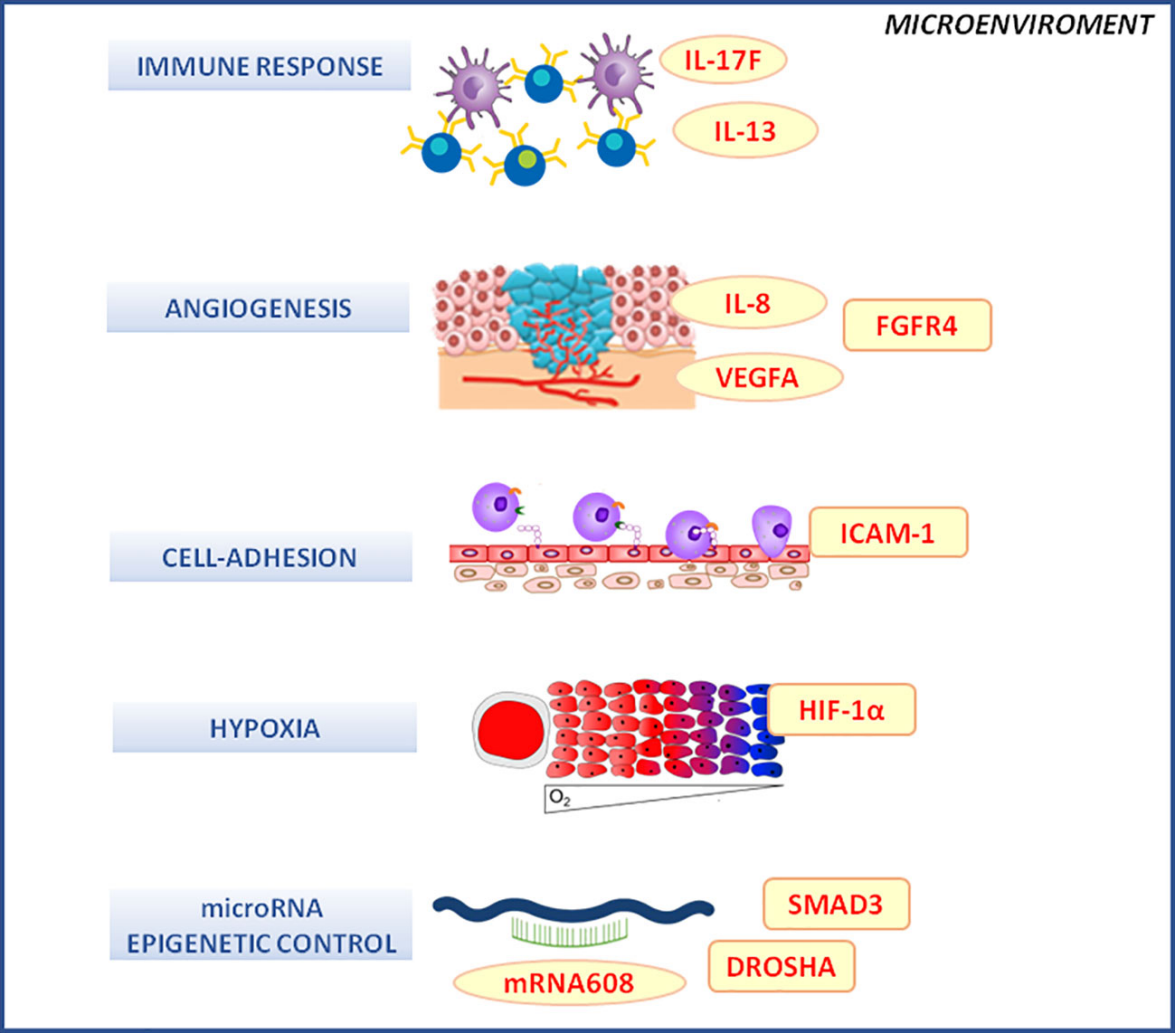
Microenvironment molecular pathways associated with the clinical outcome of neo-adjuvant chemoradiotherapy in locally advanced rectal cancer patients. DROSHA, double-stranded RNA-specific endoribonuclease; FGFR4, fibroblast growth factor receptor 4; HIF-1α, hypoxia inducible factor 1 subunit alpha; ICAM-1, intercellular adhesion molecule 1; IL-1, -6, -13, -17F, interleukin -1, -6, -13, -17F; mRNA, microRNA; SMAD-3, SMAD family member 3; VEGF, vascular endothelial growth factor.

The role of the tumor microenvironment in the response to nCRT in LARC was largely explored (Perez-Ruiz and Berraondo, 2016; Zhang et al., 2019). In this context, germline polymorphisms of proteins mediating inflammatory, angiogenic, hypoxia and cell adhesion phenomena were considered (Garziera et al., 2015; Garziera et al., 2017; Labriet et al., 2017; De Mattia et al., 2018a; De Mattia et al., 2018b; De Mattia et al., 2019; Labriet et al., 2019) (**Figure 3**). This is certainly a promising field of investigation in pharmacogenomics and this is proved by the number of studies published up to date analyzing different players and mediators of the complex inter-play between the microenvironment and the tumor tissue in determining the tumor response phenotype. However, despite many interesting results, no final consensus was reached up to date on the predictive role of each polymorphism. A lot of markers discussed below were investigated by single studies and lack of formal independent replication of the results prevents the possibility to draw final conclusion.

The promoter polymorphism rs4073 of interleukin 8 (*IL-8*), an interleukin with an important role in the angiogenic process, was linked to an increased IL-8 expression *in vitro* and was associated with a higher risk of LARC recurrence (Gordon et al., 2006). Another angiogenesis-related marker, the rs2010963 variant, located in the 5'UTR of *vascular endothelial growth factor (VEGFA)* gene and predictive of the protein serum level, was suggested to influence the probability to get a complete pathological response (Dreussi et al., 2016a). The missense variant rs351855 (Gly388Arg) in the gene encoding for fibroblast growth factor receptor 4 (FGFR4), a receptor tyrosine kinases involved in several cellular activities as angiogenesis, cell motility and inflammation, was also reported to contribute in determining the recurrence risk after radiotherapy by interacting with other genetic variants as *IL-8*-rs4073, *intercellular adhesion molecule 1 (ICAM-1)-*rs5498, and *TGFB1*-rs1800470 (Gordon et al., 2006). Among these markers, rs5498 is a missense variation (Glu469Lys) of *ICAM-1*, a cell adhesion molecule involved in the cell-to-cell interactions, another biological action under the control of the tumor microenvironment that deserves to be further investigated in the context of nCRT response in LARC.

Genetic polymorphisms in NFKB1, a key transcriptional factor for the activation of the inflammatory signaling cascades, has been also investigated. The *NFKB1* rs28362491-DEL allele, alone or in haplotype combination, was reported to be predictive of an increased rate of pathological complete response. An association trend was also observed between the rs28362491-DEL allele and a longer DFS and OS (Dzhugashvili et al., 2014). This preliminary finding suggests how an altered triggering of the inflammatory response could be related to the resistance to treatment. *TGFB1*-rs1800470 and *prostaglandin-endoperoxide synthase 2* (*PTGS2)*-rs20417 are other inflammatory-related genetic markers that have been suggested to contribute in determining the response to chemoradiation treatment in term of recurrence rate (Dreussi et al., 2016a). IL-13 is involved in the modulation of the immune system and the tumor immunosurveillance, which polymorphisms were related to the outcome of LARC patients treated with nCRT. The T-allele of the rs1800925 variant, located in the promoter region of *IL13*, was shown to be associated with a poorer response to nCRT in a cohort of Caucasian LARC patients (Ho-Pun-Cheung et al., 2011). The polymorphic T-allele was described to increase the transcription of *IL-13* that is involved in a down-regulation of tumor immunosurveillance. This is in line with the hypothesis that an impaired chemoradiation-induced tumor immunosurveillance could decrease the efficacy of the chemoradiotherapy. However, the impact of rs1800925 variant on response to radiation therapy was not confirmed in another investigation performed in a cohort of patients with LARC of Chinese ethnicity (Xiao et al., 2016). Since the genotype frequency of rs1800925 varies significantly by ethnicity, further studies are required to better clarify the predictive value of this marker in the different ethnic groups.

Our group recently produced promising data for some polymorphisms (i.e., 3'UTR rs641701, 5' UTR rs9463772) in the IL-17F encoding gene. IL-17 is an effector of the immune system that displays anti-tumor proprieties by acting on tumor angiogenesis and by improving the host inflammatory response against neoplastic cells. IL-17F rs641701-C and rs9463772-A alleles were associated with a poor prognosis in term of higher risk of disease recurrence after surgery, of distant failure of the treatment, and of death (Cecchin et al., 2020). Within a subgroup analysis, the two polymorphisms seemed to identify subcohorts of patients with a poorer long-term prognosis within homogeneous TRG strata, making them suitable to be integrated with already available prognostic clinical parameters. A hypoxic microenvironment represents another factor that was suggested to cause both a deficiency in DNA repair and a genetic instability.

The hypoxia inducible factor 1 subunit alpha (HIF-1α, encoded by HIF1A) is an important mediator of hypoxia-induced radio-resistance. The association between hypoxia-related markers (i.e., HIF1A rs11549465, rs11549467, and rs2057482 polymorphism) and response to nCRT was analyzed but no significant association were highlighted (Havelund et al., 2012).

Germline variants impacting the activity of microRNAs, with a potential epigenetic control on the overall cellular gene expression pattern were also investigated. A single variation affecting the microRNAs activity could have a downstream down-regulation effect on a large number of genes, including those involved in crucial pathways for the response to radiotherapy as DNA repair, angiogenesis, and inflammation (Dreussi et al., 2012). The work of Dreussi et al., (2016b) evaluated a set of miRNA-related TagSNPs, potentially affecting miRNA maturation and activity, in a cohort of 270 Caucasian patients with LARC stratified in two subgroup according to the radiation dose (50.4Gy or 55.0Gy). *SMAD family member 3 (SMAD3)-*rs744910, *SMAD3*-rs745103, and *trans-activation-responsive RNA-binding protein (TRBP)-*rs6088619 were associated to an increased chance of pCR, while *double-stranded RNA-specific endoribonuclease (DROSHA)*-rs10719 and *SMAD3*-rs17228212 had an opposite detrimental effect on pathological tumour response. A classification and regression tree analysis highlighted that specific combination of *SMAD3*-rs744910 and *TRBP*-rs6088619 genotypes together with clinical features (i.e., longer interval time between the end of radiotherapy and surgery) increases the chance of pCR. The finding of three independent variants (i.e., located in different haploblocks) in *SMAD3* provides a strong support for the involvement of this protein in the response to chemoradiotherapy. Another study (Sclafani et al., 2016) focused instead on a specific polymorphism, the rs4919510 C to G substitution that affects the mature microRNA 608 previously associated with response to treatment in patients with CRC. Within a retrospective analysis of the EXPERT-C phase II trial, the rs4919510-CC genotype was associated with worse 5 years PFS and OS after neo-adjuvant CAPOX followed by capecitabine-based chemoradiotherapy. The polymorphism probably impacts the interaction between miR-608 and its target mRNAs in a tissue-specific manner.

Most of the pharmacogenomic studies that investigated the response to nCRT in LARC have adopted a candidate gene or a pathway-based approach. Alternatively, two works opted for an unbiased strategy, performing a genome-wide analysis including thousands of polymorphisms. The study of Kim and colleagues (Kim et al., 2013), including 113 Korean patients with LARC receiving FP-based CRT, implemented a 3-step genome-wide strategy, based on genome-wide screening, clinical association, and biological validation of predictive polymorphisms. At the end, two novel markers were identified as potential predictors of response to treatment, *coronin 2A (CORO2A)* rs1985859 and *refilin A (FAM101A*) rs7955740. The reference *CORO2A* rs1985859-C allele was associated with higher rate of positive response. Moreover, an *in vitro* assay highlighted that the downregulation of CORO2A, linked to the variant rs1985859-T allele, was associated with reduced early apoptosis, increased cell survival or viability, and lower radiosensitivity. Even if *FAM101A* rs7955740 was not related with therapy outcome in the clinical association study, its downregulation, linked to the minor G-allele, was associated with a reduced early apoptosis and lower radiosensitivity similarly to *CORO2A* rs1985859. Another study (Lee et al., 2018), including a similar cohort of Korean patients with LARC and adopting a discovery/validation design, performed a whole-exome sequencing analysis identifying some further potential novel markers. Overall, five candidate variants emerged, *Bcl-2-like protein 10 (BCL2L10)* rs2231292, *DLC1 Rho GTPase activating protein (DLC1)* rs3816748, *dynein axonemal heavy chain 14 (DNAH14)* rs3105571, *inter-alpha-trypsin inhibitor heavy chain 5 (ITIH5)* rs3824658, and *retinoic acid early transcript 1L (RAET1L)* rs912565. Particularly, *DLC1*-rs3816748-C allele, *DNAH14* rs3105571-C allele, and *RAET1* rs912565-TT genotypes were associated with a higher rate of pCR according to a dominant model, while *BCL2L10* rs2231292-CC genotype and *ITIH5* rs3824658-T allele according to recessive model. In the co-dominant model, four candidate variants (all except *BCL2L10* rs2231292) were significantly correlated with pCR. The identified polymorphisms potentially impact the functionality of the encoded proteins that are involved in crucial biological pathway as tumor suppression, transport along microtubules, and regulation of cell apoptosis, extracellular matrix stability, tumor invasion and metastasis. The markers of radiosensitivity emerged from genome-wide analyses, could represents an interesting candidate that require to be validated by larger independent studies.

## Somatic Pharmacogenomic Profile

Several studies investigated the potential predictive role of some somatic mutations in patients with LARC treated with a nCRT but their predictive/prognostic value in this setting remains uncertain. The most studied candidate genes are those belonging to PI3K cancer-related pathway, and *KRAS* in particular, due to its primary role in colorectal cancer. KRAS plays a major role in two tumor-related cellular pathways, mitogen activated protein kinase (MAPK) and PI3K/AKT, by regulating their activation in response to cellular stimuli. Mutant forms of KRAS can cause a stable activation of those cellular pathways conferring a more aggressive tumor phenotype and resistance to anti-EGFR agents as cetuximab or panitumumab.

To this regard a meta-analysis published in 2013, analyzing the results of seven studies published between 2010 and 2012, concluded that somatic mutations in *KRAS* were neither predictive nor prognostic pharmacogenomic markers in LARC patients treated with nCRT (Clancy et al., 2013). Going deeply in the aforementioned studies, we could notice that just two of them included more than 100 patients, that the pharmacological treatments received were quite heterogeneous with three out of seven studies including cetuximab as a co-treatment, and that the frequency of *KRAS* mutation was lower than expected, probably due to the use of low sensitivity genotyping techniques. The last observation could be related to the sequencing method used (Sanger direct sequencing approach), sometimes focused only on hot-spot regions.

A number of studies have been conducted and published afterwards providing some more convincing evidence of an actual role of somatic mutations in *KRAS* and other related genes [*NRAS proto-oncogene, GTPase (NRAS), B-Raf proto-oncogene, serine/threonine kinase (BRAF)*, and *PIK3CA*] in the identification of low responder/bad prognosis patients. Most recent studies are characterized by the use of more sensitive DNA sequencing/genotyping approaches that led to the identification of a higher rate of tumors with mutation in *KRAS*, with an average frequency of about 40% of patients carrying at least one somatic mutation. Moreover, larger studies including a higher number of patients and combining the *KRAS* genetic information with other relevant genetic alterations in the same pathway or in *tumor protein p53 (TP53)* gene, allowed to provide a better idea of the actual predictive/prognostic role of somatic pharmacogenomics in LARC.

In 2013 a study by Duldulao and colleagues (Duldulao et al., 2013b) reported the results of a prospective multicenter clinical trial investigating the effect of increasing the nCRT-to-surgery time interval and adding chemotherapy during the waiting period (ClinicalTrials.org Identifier: NCT00335816). In a group of 148 stage II-III rectal cancer patients undergoing *KRAS* and *TP53* genotyping on pretreatment tumor biopsies, they demonstrated that patients with *KRAS* mutated tumors had a decreased chance to get a pathological complete response compared to wildtype *KRAS* tumors, and specifically, no tumor with a KRAS mutation in codon 13 had a complete response.

The following year, another group (Abdul-Jalil et al., 2014) performed the genotyping of 234 potentially clinically relevant nonsynonymous mutations in 33 PI3K and MAPK pathway-related genes, including *PIK3CA, PIK3R1* (encoding phosphoinositide-3-kinase regulatory subunit 1)*, AKT, STK11* (encoding serine/threonine kinase 11)*, KRAS, BRAF, MEK* (encoding mitogen-activated protein kinase kinase)*, CTNNB1* (encoding catenin beta 1), EGFR, MET (encoding MET proto-oncogene, receptor tyrosine kinase), and NRAS, using the Sequenom platform on pretreatment LARC biopsy samples from 201 patients with LARC treated with nCRT. Patients without mutations in PI3K pathway-related genes, thus including RAS/RAF, were more likely to have pCR after nCRT. The same association was not observed for mutations in MAPK pathway-related genes.

In 2016, Chow and colleagues (Chow et al., 2016) published the results of a retrospective analysis performed on 229 pretreatment biopsies from patients with stage II/III rectal cancer receiving FOLFOX (i.e., folinic acid, fluorouracil, and oxaliplatin) treatment either before or after nCRT, but prior to surgical excision. Tumor DNA samples were sequenced to highlight the presence of either *TP53* or *KRAS* mutations. It was demonstrated that 34% of patients with a *KRAS* wild-type tumors had a pCR in comparison to 15% of *KRAS* mutated tumors (p=0.001). When specifically focusing on *KRAS* Gly12Val or Gly13Asp mutations, the percentage of complete responders lowered to 7%. When considering the combination of mutations in both *KRAS* and *TP53* in the same tumor, the risk of lymph node metastasis was also increased.

More recently a comprehensive and well conducted study was published by Sclafani et al. (2020) reporting on 210 patients with LARC treated with neoadjuvant CAPOX (i.e., capecitabine and oxaliplatin) followed by capecitabine-based chemoradiotherapy with or without cetuximab based on a prospective clinical trial (PAN-EX study). The mutational status of *KRAS, NRAS, BRAF, PIK3CA*, and *TP53* was assessed on the pre-treatment biopsy. The presence of *TP53* mutations was a risk factor for extramural venous invasion, poor pathological response and 5-year PFS. Even if similar validated data are not up to date available for *LARC*, very promising results have been published about the role of the same molecular markers, and specifically of somatic mutation in *KRAS* and *TP53*, as predictors of tumor response and prognosis. Tumors simultaneously carrying a mutation in *TP53* and either *KRAS* or *NRAS*, had a worse prognosis than those with a *TP53/KRAS/NRAS* wild genotype. This study suggests how integrating different molecular markers could better refine the predictive/prognostic value of specific pharmacogenomic features of patients with LARC. Other examples of such an approach can be found in the study by Kamran et al., (2019) that in a small set of well-studied LARC, treated with nCRT demonstrated that concurrent *KRAS/TP53* mutations were associated with a non-responder tumor phenotype and were enriched for an epithelial–mesenchymal transition transcriptional profile.

Another small study by Krajnovic and colleagues (Krajnovic et al., 2016) investigated the mutational status of *KRAS* gene in the pre-treatment biopsies of 63 patients with LARC. The genetic information was integrated with the immunohistochemical analysis of VEGF and Ki67. Even if *KRAS* mutation status by itself was not predictive of any clinical outcome evaluated, including pathological response, a simultaneous high VEGF expression was related to worse response to nCRT, higher risk of local recurrences and distant metastasis, and shorter OS. The predictive effect of the combination of somatic mutations in *BRAF* and *SMAD family member (SMAD4)* was investigated by Jiang and colleagues in 2019 (Jiang et al., 2019) in 74 patients with LARC treated with a FP-based induction chemotherapy followed by nCRT. It was demonstrated that *BRAF* and *SMAD4* were more frequently mutated among non-responder patients, and that the mutations were negative prognostic factors.

Interestingly some studies reported, as previously observed for colorectal cancer, a specific role of different *KRAS* somatic mutations, suggesting that a different contribute to tumor aggressiveness and treatment sensitivity could derive from different alterations in the *KRAS* protein structure. The previously mentioned study by Abdul-Jalil et al. (2014) reported that in patients not achieving a pCR, only mutations in codon 12 (Gly12Asp/Gly12 Val/Gly12Ser) and codon 13 mutations in *KRAS* were associated with poor recurrence-free survival. Gaedke and colleagues (Gaedcke et al., 2010) demonstrated that in 94 patients with LARC, tumors bearing a Gly12Val mutation were related to significantly higher rates of tumor regression than those with a Gly13Asp mutation, despite the overall presence of *KRAS* mutations did not correlate with tumor response and patient's outcome after preoperative chemoradiotherapy. In the previously mentioned study by Krajnovic et al. (2016) patients with a Gly12Ala mutation in *KRAS* had a significantly improved tumor response to CRT than those with any other type of *KRAS* mutation. Despite the heterogeneity of these results, probably driven also by the low numerosity of patients in each subgroup, it appears evident that not all mutations have the same value when considering the impact on the tumor phenotype and specific studies should be performed in order to assess the predictive/prognostic value of each mutation.

Most recent studies highlighted that in the oncological setting the immune system could play a pivotal role also in the tumor response to nCRT in patients with LARC. In one of the above mentioned study, published in 2019 by Kamran and colleagues (Kamran et al., 2019), 34 pre- and post-nCRT–matched tumor samples from 17 patients with LARC who received FP-based nCRT, followed by surgical resection, were analyzed. The authors demonstrated that the non-responder phenotype was associated with reduced CD4/CD8 T-cell tumor infiltrates and with a post-CRT M2 macrophage phenotype. These results highlighted how local tumor immune escape together with specific genomic features can contribute to the efficacy of chemoradiotherapy in the control of distant disease progression, paving the way to new therapeutic approaches and a new generation of predictive/prognostic markers in the neo-adjuvant treatment of LARC.

## Conclusion and Future Perspectives

Important refinements to the patients' stratification based on clinical, pathological and radiological parameters have been reached. Recently, the advanced analysis of medical images and their correlation with patient's outcome, using machine learning techniques (radiomics) were demonstrated to be helpful in predicting tumor response to pCRT in LARC (Dinapoli et al., 2018; Pirrone et al., 2019). Still significant inter-individual differences in patient's outcome, not captured by currently employed risk algorithms solely based on patients clinicopathological risk features are observed. Despite extensive research programs developed with the aim of identifying new criteria to adapt neo-adjuvant treatment programs based on the patient's molecular profiles, still too little is known about the predictive/prognostic effect of germline and somatic pharmacogenomic variants.

Considering that FP represent the backbone of each nCRT regimen in LARC, the most relevant drug-gene interaction that should be considered when planning a treatment is related to the study of *DPYD* polymorphisms in the context of increasing treatment safety. The clinical impact of testing the four polymorphisms panel (DPYD*2A, DPYD*13, *c.2846A > T*, and *c.1236G > A-HapB3)* on reducing the risk to develop severe toxicity and the costs associated to the treatment of the adverse events has been fully elucidated. The test still struggles to be integrated in the routine clinical practice of oncological treatments. However, the available pharmacogenetic guidelines on *DPYD* testing have been recently endorsed by some international regulatory agencies, as European Medicines Agency, that recently recommended to perform the test prior a FP administration. This will probably represent the ultimate step towards the clinical implementation of this life-saving analysis in the clinical management of LARC and of many other human malignancies.

In the context of FP treatment, other gene-drug interactions have been studied with the aim to improve treatment efficacy, by the analysis of polymorphisms in *TYMS* and *MTHFR*. The identification of germline genetic markers predictive of tumor response or prognosis is in general more difficult to be accomplished due to the complexity of the tumor response phenotype and to the contribution of the tumor genome that could significantly differ from the germline. A pivotal role of the *TYMS* polymorphism in the gene promoter region (*TSER*, rs34743033) has been reported by several studies, demonstrating that alleles associated with an increased target protein expression seems to lower the efficacy of a FP based treatment. The interventional phase II study based on those evidences demonstrated that the *TSER* polymorphism may be used as a baseline selection criterion in LARC patients to personalize nCRT by chemotherapy intensification in patients with a high TYMS expressing tumors. The study published in 2011 was not followed by further similar experiences at our knowledge. The validation of the prospective use of TSER, possibly in combination with other more recently developed predictive markers could represent a further step towards a personalization of LARC multimodality treatment. As far as *MTHFR* is concerned, several studies were concordant in assessing a detrimental role of the rs1801133 polymorphism on patients with LARC tumor response and survival and further effort should be made to test its effect in prospective interventional studies in order to eventually define its clinical applicability.

More exploratory data are available on other cellular pathways that demonstrated a primary role in the clinical outcome of patients undergoing chemoradio combination treatments as DNA repair and inflammation. Those pathways, that control the repair of radiation generated damage and the consequent activation of the inflammatory cascade, represent an important source of inter-individual variability in the outcome of nCRT. Most consistent data are related to the role of polymorphisms in *TGF-β* (particularly rs1800471) on the risk of developing treatment-related toxicity and a differential chance of tumor response. The anti-tumor effect of ionizing radiation is known to be linked not only to a direct damaging effect on tumor cells DNA and to the generation of free oxygen radicals, but also to the priming effect of radiotherapy on the immune system. Administration of nCRT treatment in patients with LARC can significantly modify the tumor tissue immune-profile and stimulate the release of cancer derived neo-antigens, resulting in the so-called “immunogenic cell death”. The effect of radiotherapy on the activation of an immunogenic cytotoxic effect is mediated by a complex network of cytokines and chemokines that are released in the tumor microenvironment attracting dendritic cells. By reviewing literature, it appeared that genetic polymorphisms in those immune mediators can affect the risk of toxicity, tumor response and patients prognosis. Genetic polymorphisms in DNA repair genes as *XRCC1, ERCC1*, and *RAD51* have been studied for a long time as potential germline markers of radio sensitivity. Nonetheless, even if an overall effect of these genes is likely, no candidate markers with a promising application in the patients with LARC treatment scheduling is currently identifiable.

Globally, the results of available pharmacogenetic studies on germ-line predictive markers in patients with LARC published over the past 10-years have produced findings that in the majority of the cases did not allow a translation in the clinical practice, suggesting that a critical revision of research strategies is required. The impossibility to demonstrate a strong clinical validity of the investigated markers was in most cases the primary reason that preventing them to be transferred to the clinical practice. The high heterogeneity among published studies and the low number of patients included surely account for part of the difficulty to compare data and find reliable markers of clinical utility. Future investigations aimed at discover solid predictors of radio sensitivity should be performed on larger cohorts of patients homogeneous for ethnicity and treatment modalities, including dosages, schedules and formulations used for radiotherapy and drugs administration (e.g., total radiation and FP dose; administration of 5-FU or capecitabine that presents different radio-sensitizing activity; other co-administered chemotherapeutics, as platinum derivates, irinotecan, cetuximab; time frames between preoperative therapy end and surgery). Standardization of the clinical monitoring strategies, clinical end-point assessment (i.e., clinical endpoints and methods to evaluate and score tumor response), as well as genotyping methods are additional aspects to be standardized (Di Francia et al., 2010).

The effect of the somatic pharmacogenomic profile was extensively investigated in advanced colorectal cancer and in stage II-III colon cancer, and the clinical significance of mutations in cancer-related genes such as *BRAF, KRAS, NRAS*, and *TP53* is widely acknowledged. The analysis of the mutational status of *RAS/RAF* genes represents a mandatory test in the metastatic colorectal cancer to assess tumor sensitivity to anti-EGFR monoclonal antibodies. Even if similar validated data are not currently available for LARC, very promising results were published about the role of the same molecular markers, and specifically of somatic mutation in *KRAS* and *TP53*, as predictors of tumor response and prognosis. The somatic pharmacogenomic profile of the tumor represents a useful piece of information in the oncologist toolbox that can be helpful in the outline of the most appropriate therapeutic approach also in this clinical context.

Indeed, the tumor genomic landscape is not stable and the disease evolution, as well as the selective pressure of the radio and chemotherapy can modify its characteristics. In this context a dynamic monitoring of the somatic genomic profile could anticipate the occurrence of tumor pharmaco-resistant or more aggressive clones overcoming. The discovery of the presence of circulating tumor DNA (ctDNA) in patients' blood and the possibility to analyze it through digital next-generation sequencing technologies led to important advances in the field. Several studies currently demonstrated a good correlation between the information derived from tumor tissue DNA sequencing and circulating tumor DNA (Coombes et al., 2019; Mezzalira et al., 2019; Siravegna et al., 2019; Dalle Fratte et al., 2020) paving the way to the use of ctDNA as a new candidate biomarker to be used either as a diagnostic or as a predictive/prognostic tool in cancer.

A number of researchers investigated the role of ctDNA as a biomarker of the therapeutic outcome in LARC and the results were recently reviewed by Massinhia and colleagues (Massihnia et al., 2019). Particularly a study by Tie et al. (2019) analyzed in a large group of 159 patients with LARC by sequencing cell free DNA in serial plasma samples at the time of diagnosis, after nCRT and after surgery demonstrating that the tumor DNA fraction level in plasma was significantly related to the risk of treatment failure. Most recently Khakoo et al., (2020) demonstrated how an integrated approach integrating standard clinical monitoring of tumor response with information deriving from the study of ctDNA in plasma can better identify patients at risk of developing metastases after surgery. This tool could represent an easily translatable test in the clinical practice helping the selection of patients for more conservative surgical approaches or less invasive therapeutic options after surgery.

In conclusion, despite the advancements in the field of personalized medicine and availability of treatments based on specific molecular targets, still the great molecular heterogeneity among patients and tumors represents a barrier to reach the final goal of a precision medicine in oncology. Different predictive methods have been explored, but none of these has showed enough accuracy to be used in the clinical setting. This is likely due to the high level of heterogeneity of the disease that considers complex interaction of genetic, molecular, and physiological features. The application of germline and somatic pharmacogenomics in the context of nCRT in LARC has provided up to date few validated markers as *DPYD* polymorphisms for preventing toxicity and *TYMS-TSER* or *KRAS* somatic variants to identify poor responders to treatment. A number of additional germline and somatic markers are under investigation and have already demonstrated encouraging clinical evidence of predictive and prognostic value and are likely to get in the future a clinical application. To identify in advance the toxicity and efficacy outcome to nCRT is an “unmet clinical need” in LARC to avoid under- or over-treatment. This would result into an optimal treatment approach, increasing treatment efficacy, minimizing surgery-related morbidity, avoiding unnecessary side-effects, improving quality of life, and reducing healthcare costs.

## Author Contributions

EM performed the literature review and analysis and contributed to writing the manuscript. RR contributed to writing the manuscript and elaborated the tables. EP contributed to writing the manuscript. GT edited the manuscript. EC conceptualized and contributed to writing the manuscript.

## Funding

This work was supported by the European Union's Horizon 2020 Research and Innovation Program (grant agreement no. 668353) (Ubiquitous Pharmacogenomics- UPGx).

## Conflict of Interest

The authors declare that the research was conducted in the absence of any commercial or financial relationships that could be construed as a potential conflict of interest.

## Abbreviations

5-FU, 5-fluorouracil; AKT, v-akt murine thymoma viral oncogene homolog; AKT,v-akt murine thymoma viral oncogene homolog; APE1, apurinic/apyrimidinic endodeoxyribonuclease 1; AREG, amphiregulin; ATM, ATM serine/threonine kinases; ATR, ATR serine/threonine kinases; BCL2L10, Bcl-2-like protein 10; BER, base excision repair; BRAF, B-Raf proto-oncogene, serine/threonine kinase; CCDN1, cyclin D1; CORO2A, coronin 2A; COX-1, -2, cycloxygenase-1, -2; ctDNA, circulating tumor DNA; DFS, disease-free survival; DLC1, DLC1 Rho GTPase activating protein; DNAH14, dynein axonemal heavy chain 14; drosha, double-stranded RNA-specific endoribonuclease; DSB, double-strand breaks; DYPD (encoding DPD), dihydropyrimidine dehydrogenase; EGFR, epidermal growth factor receptor; ERCC1, -2, ERCC excision repair 1, -2; FAM101A, refilin A; FGFRs, fibroblast growth factor receptors; FP, fluoropyrimidine; GST, glutathione S-transferase; HIF1A (encoding HIF-1α), hypoxia inducible factor 1 subunit alpha; hOGG1, 8-oxoguanine DNA glycosylase; HR, homologous recombination; ICAM-1, Intercellular Adhesion Molecule 1; IL-X, interleukin-X; ITIH5, inter-alpha-trypsin inhibitor heavy chain 5; KRAS, KRAS proto-oncogene, GTPase; LARC, locally advanced rectal cancer; MAPK; mitogen activated protein kinase; MDM2, MDM2 proto-oncogene; MGMT, O6-methylguanine DNA methyltransferase; MLH1, mutL homolog 1; MPO, myeloperoxidase; MSH6, mutS homolog 6; MTHFR, methylenetetrahydrofolate reductase; mTOR, mammalian target of rapamycin; nCRT, neoadjuvant chemoradiation; NER, nucleotide excision repair; NFKB, nuclear factor κB; NHEJ, non-homologous end joining; NOS3, nitric oxide synthase 3; NRAS, NRAS proto-oncogene, GTPase; OS, overall survival; PAI-1, plasminogen activator inhibitor type 1; PAR-1, protease-activated receptor-1; PFS, progression-free survival; PI3K, phosphoinositide-3-kinase; PI3KCA, phosphoinositide-3-kinase catalytic subunit alpha; PTEN, phosphatase and tensin homolog; PTGS-1, -2, prostaglandin-endoperoxide synthase 1, - 2; RAD51, RAD51 recombinase; RAET1L, retinoic acid early transcript 1L; ROS, reactive oxygen species; RT, radiotherapy; SMAD-3, SMAD family member 3; SOD2, superoxide dismutase 2; TGF-β1, transforming growth factor beta 1; TNFA (encoding TNF-α), tumor necrosis factor-alpha; TP53 (encoding P53), tumor protein p53; TP73, (encoding P73) tumor protein p73; TRBP, trans-activation-responsive RNA-binding protein; TYMS (encoding TS), thymidylate synthase; UTR, untranslated; VEGF, vascular endothelial growth factor; VEGFR, vascular endothelial growth factor receptor; XPD, -A, xeroderma pigmentosum group D, -A; XRCC1, -3, x-ray repair cross-complementing 1, -3.
