# Supplementary material for: Germline and Somatic Pharmacogenomics to Refine Rectal Cancer Patients Selection for Neo-Adjuvant Chemoradiotherapy
Source: Front Pharmacol. 2020 Jun 17;11:897. doi: 10.3389/fphar.2020.00897 (PMC7311751; doi:10.3389/fphar.2020.00897)
Supplement: Supplementary file 1 [file Table_1.docx]

**Supplementary Table 1: Published works on germ-line variants and toxicity in locally advanced rectal cancer (LARC) patients receiving neoadjuvant chemoradiotherapy (nCRT)**

| **Pharmacogenetic panel** | **Study Population** | **Neo-adjuvant regimen** | **Clinical End-point** | **Main findings** | **Other findings** | **Reference** |
| --- | --- | --- | --- | --- | --- | --- |
| **MTHFR** | | | | | | |
| 10 variants in 6 genes (TYMS, MTHFR, DPYD, UGT1A1, ABCC1 and SLCO1B1). | 131 LARC patients (Caucasian, USA) | CT: 5-FU-based ± IRI (IRI n=35 patients, non-IRI n=96).  RT: Total dose of 45–50.4Gy | Toxicity (NCI-CTCAE v.2.0); no association observed on drug response. | MTHFR rs1801131 and MTHFR diplotypes (for rs1801133 and rs1801131) were associated with toxicity when 5-FU was used alone (OR:4.71, *P*=0.005). MTHFR haplotypes (rs1801133-C/rs1801131-C) and diplotypes (CA–TA and TA–TA) showed, respectively, a protective (OR:0.21, *P*=0.005) and a detrimental effect on the incidence of severe diarrhea or mucositis (OR:7.75, *P*=0.003). |  | Thomas et al., 2011 |
| **DPYD** | | | | | | |
| 4 SNPs in DPYD (DPYD*2A, c.1905þ1G>A, rs3918290; DPYD*13, c.1679T>G, rs55886062; c2846A>T, rs67376798; c.1236G>A/HapB3, rs56038477). | 93 LARC patients | CT: FPs-based (CAPE n=92; 5-FU n=1)  RT: 55Gy median RT | Toxicity (NCI-CTCAE v3.0 or v4.03) | DPYD variant allele carriers:   - treated with standard dosages had an increased risk of severe gastrointestinal (adj OR:2.58, *P*=0.045) or severe hematological (adj OR:4.19, *P*=0.015) toxicity compared with WT patients. - treated with dose reductions had a comparable frequency of severe gastrointestinal toxicity compared with WT patients, but more (not statistically significant) severe hematological toxicity. Mean duration of hospitalization was significantly shorter in the dose reduction group. |  | Lunenburg et al., 2018 |
| **DNA repair** | | | | | | |
| XRCC1-rs25487; XRCC3-rs1799794, -rs861539; RAD51-rs1801320; GSTP1-rs1695. | 67 LARC patients  (Caucasian) | CT: FPs-based (5-FU or CAPE)  RT: Total dose of 45.0Gy | Acute Toxicity  (NCI-CTCAE v.3.0) | - RAD51 rs1801320-C allele (vs GG) associated with increased grade ≥3 abdominal/pelvis pain toxicity (22.2% vs 3.4%, *P*=0.027) and acute skin toxicity of any grade (55.6% vs 22.8%, *P*=0.04). - XRCC1 rs25487-A (Gln) allele (vs GG) associated with increased skin toxicity of any grade (36.6% vs 12%, *P*=0.03). - XRCC3 rs1799794-GG (25%, *P*=0.01), AG (12.5%, *P*=0.022), and GG/AG (11.1%, *P*=0.031) associated with increased grade ≥3 urinary frequency/urgency; rs1799794-GG associated with higher risk of acute skin toxicity of any grade (75% vs 25%, *P*=0.015). XRCC3 rs1799794-AG (vs AA) (29.2% vs 10%, *P*=0.049) associated with higher rates of fatigue. |  | Osti et al., 2017 |
| 22 SNPs in 17 genes (ERCC1, XRCC1, RAD23B, XPA, ERCC2, hOGG1, PARP, MTHFR, TS, DPYD, OPRT, CCND1, TP53, EGFR, VEGF, IL6, TLR2). | 132 LARC patients  (mixed, mainly Caucasian) | 5-FU-based ± modified FOLFOX-6 CRT. | Toxicity  (NCI-CTCAE v.3.0) | - XRCC1 rs25487-A (Gln) allele (24% vs 5%, *P*=0.01) and ERCC2 rs13181-AA (Lys/Lys) genotype (27% vs 12%, *P*=0.02) associated with increased grade ≥3 toxicity. - TP53 rs1042522-C (Pro) (26% vs 5%, *P*=0.008) associated with increased rate of grade ≥3 toxicity to modified FOLFOX-6. |  | Duldulao et al., 2013a |
| **Inflammation** | | | | | | |
| XRCC1- rs25487; ERCC2- rs13181; TGFB1-rs1800471 | 165 LARC patients  (Caucasian, 82%; Black, 8%; Asian, 10%) | CT: FPs-based (5-FU or CAPE)  RT: Total dose of 50.4Gy | Acute Toxicity  (classified by NCI-CTCAE v.3.0 system, n=165; by also 7-item Bowel Problems Scale, n=52) | - TGFB1 rs1800471-C (Pro25) allele (vs GG) associated with increased grade ≥3 toxicity (OR:3.47, *P*=0.04) and, in patients who completed the Bowel Problems Scale, with grade ≥4 toxicity (OR:5.61, *P*=0.02). Confirmed in multivariate analysis (OR:1.83, *P*=0.02). | - XRCC1 rs25487-A (Gln) allele (vs GG) showed an association trend with grade ≥3 toxicity (OR:4.25, P=0.06) | Smith et al., 2017 |
| TNFA-rs1799964, -rs1800629; IL6-rs1800796; IL1-rs1143623, -rs1143627 | 356 rectal cancer patients  (Chinese) | Concurrent CT: 7.6%, none; 62.6% 5-FU or CAPE combined with OXA; 10.1%, IRI and CAPE; 19.7%, single drug (5-FU or CAPE)  RT: Total dose ≤50Gy (61.5%); >50Gy (38.5%). Pelvic RT: 58.8%, pre-operative; 32.4% post-operative; 8.8%, recurrent | Acute Toxicity  (NCI-CTCAE v.3.0) | - TNFA rs1799964-T allele was associated with increased risk of total grade ≥2 acute toxicity (CT vs CC: adj OR:4.718, *P* = 0.031; TT vs CC: adj OR:4.443, *P*=0.034; CT/TT vs CC, adj OR:4.132, *P*=0.04). - *In vitro* analysis suggested that the rs1799964-T allele was associated with higher TNF mRNA expression levels in Chinese population. |  | Zhang et al., 2015a |
| 6 SNPs in PAI-1 and PAR-1 genes | 356 rectal cancer patients  (Chinese) | CT: 7.6%, none; 62.6% 5-FU or CAPE plus OXA; 10.1%, IRI and CAPE; 19.7%, single drug (5-FU or CAPE)  RT: Total dose ≤50Gy (61.5%); >50Gy (38.5%). Pelvic RT: 58.8%, pre-operative; 32.4% post-operative; 8.8%, recurrent | Acute Toxicity  (NCI-CTCAE v.3.0) | - PAI-1 rs1050955-GG genotype (vs AA) associated with decreased grade ≥2 diarrhea (adj OR:0.394, *P*=0.019) and fecal incontinence (anal toxicity) (adj OR:0.474, *P*=0.02). - PAI-1 rs2227631-GG genotype (vs AA) associated with increased grade ≥2 incontinence (adj OR:3.172, *P*=0.003). - PAI-1 rs2227631-GG genotype (vs A-allele) associated with increased overall grade ≥2 toxicity (adj OR:1.995, *P*=0.012); PAR-1 rs32934-CT genotype (vs CC) with inferior risk (adj OR:1.778, *P*=0.037). |  | Zhang et al., 2015b |
| NFKB1-rs28362491; PTGS1-rs1213266, -rs5789; PTGS2-rs5275; IL1B-rs16944, -rs1143627. | 159 LARC patients  (Hispanic) | CT: CAPE-based  RT: Total dose of 50.4Gy | Acute Toxicity  (NCI-CTCAE v.4.0) | - NFKB1 rs28362491-DEL/DEL genotype (vs INS-allele) associated with increased grade ≥2 dermatitis (OR:3.31, *P*=0.01) and proctitis (OR:3.32, *P*=0.02). - By haplotype analysis, rs28362491-DEL/rs1143627-A/rs1213266-G/rs5789-C/rs5275-A/rs16944-G associated with increased clinically significant grade≥2 acute organ toxicity (OR:4.12, *P*=0.037), respect the reference haplotype (rs28362491-INS/rs1143627-A/rs1213266-G/rs5789-C/rs5275-A/rs16944-G). |  | Dzhugashvili et al., 2014 |
| 9 TagSNPs in TGFB1 | 163 LARC patients  (discovery cohort, n=88; replication cohort n=75)  (Caucasian) | CT: 5-FU-based ± OXA  RT: Total dose of 50.4Gy | Acute Toxicity  (i.e., QAOT*)  **defined as an NCI-CTCAE v.3.0 grade ≥2 for at least one case of enteritis, proctitis, cystitis, or dermatitis*. | In both cohorts, all patients carrying the TGFB1 rs1800471-C (Pro25) allele experienced QAOT (positive predictive value of 100%, adjusted *P*=0.0006). |  | Schirmer et al., 2012 |

Abbreviations: 5-FU, 5-fluorouracil; Adj, adjusted; CAPE, capecitabine; CRT, chemoradiotherapy; CT, chemotherapy; CTCAE, Common Terminology Criteria for Adverse Events; FOLFOX, folinic acid, fluorouracil and oxaliplatin; FPs, fluoropyrimidines; IRI, irinotecan; NCI, National Cancer Institute; OR, odd ratio; OXA, oxaliplatin; QAOT, quality of life-impairing acute organ toxicity; RT, radiotherapy; SNP, single nucleotide polymorphism; TagSNPs, tagging polymorphisms; WT, wild-type.
