# Supplementary material for: Germline and Somatic Pharmacogenomics to Refine Rectal Cancer Patients Selection for Neo-Adjuvant Chemoradiotherapy
Source: Front Pharmacol. 2020 Jun 17;11:897. doi: 10.3389/fphar.2020.00897 (PMC7311751; doi:10.3389/fphar.2020.00897)
Supplement: Supplementary file 2 [file Table_2.docx]

**Supplementary table 2: Published works on germ-line variants and efficacy in locally advanced rectal cancer (LARC) patients receiving neoadjuvant chemoradiotherapy (nCRT)**

| **Pharmacogenetic panel** | **Study Population** | **Neo-adjuvant regimen** | **Clinical End-point** | **Main findings** | **Other findings** | **Reference** |
| --- | --- | --- | --- | --- | --- | --- |
| **TYMS** | | | | | | |
| TYMS-rs34743033 (2R/3R) (TSER*2 and *3), TYMS-rs2853542 (G>C) and TYMS-rs16430 (1494del6). | **META-ANALYSIS for germ-line markers**  892 cases for TYMS 2R/3R (7 studies); 715 cases for TYMS 1494del6 (7 studies) and 616 cases for TYMS 5′UTR expression allele SNP (6 studies) | CT: FPs-based (5-FU, CAPE) plus other drugs (platinum agents, cetuximab, IRI) and RT. | TRG (TRG 1–2 or 1: responders; 3–5 or 2-5: non-responders). | Patients with TYMS 2R/2R or 2R/3R genotype with rectal cancer might benefit more from nCRT than others. On the contrary, neither 1494del6 nor 5′UTR expression allele polymorphisms were associated with the response to nCRT. |  | Yang et al., 2017 |
| TYMS-rs34743033 (2R/3R) (TSER*2 and *3), -rs2853542 (G>C) and -rs16430 (1494del6). | 99 patients LARC (Caucasian, Hispanic) | CT: 825mg ⁄m2 CAPE twice daily during the RT period (n=61) or 5-day infusion of 5-FU during weeks 1 and 5 (n=38).  RT: total dose of 45–50.4Gy | TRG (TRG1-2: good responders; TRG3-4 poor responders, Mandard system); 5-yr DFS | TYMS-rs16430 (1494del6) was significantly associated with DFS (+⁄+ vs DEL⁄DEL, *P*=0.032 logistic regression). |  | Arrazubi et al., 2013 |
| 12 SNPs in 6 genes (XRCC1, ERCC1, MTHFR, EGFR,  DPYD, TYMS). | 93 LARC patients  (Caucasian, Hispanic) | CT: 5-FU–based CRT  RT: Total dose of 50.4Gy | TRG  (TRG1-2: good responders; TRG3-5: poor responders, Mandard system) | - TYMS high expressing alleles (2R/3G, 3C/3G, and 3G/3G): Improved response rate compared (OR:2.65, *P*=0.02). | - XRCC1 rs25487-GG (Arg/Arg): better response than the AG (Arg/Gln) genotype (OR:4.180, *P*=0.003). | Lamas et al., 2012 |
| 11 SNPs in 6 genes (TYMS, ERCC1, ERCC2, XRCC1, EGFR, GSTP1). | 128 LARC patients  (Caucasian, Hispanic) | CT: FPs-based (5-FU or CAPE) ± OXA  RT: Total dose of 45Gy | TRG  (RR, pathologic complete remission and microfoci residual tumor, Mandard system)  CRM  PFS  OS | - TYMS *3/*3 genotype (vs *2/*2 or *2/*3): greater RR (59% vs 38%, *P*=0.023), longer median PSF (103 vs 75 mo., *P*=0.039) and OS (124 mo. vs 78 mo., *P*=0.02). | - XRCC1 rs25487 genotype: different median PFS (GG: 101 mo., GA: 78 mo., AA: 31 mo., *P*=0.048). - ERCC1 rs11615-TT (vs C-allele): higher rate of R1-R2 CRM resection (22% vs. 8%, *P*=0.025) and higher rate of recurrence even if not significant (TT: 42%, CT:33%, CC:24%, *P*=0.2). | Paez et al., 2011 |
| TYMS-rs34743033 (2R/3R/4R) (TSER*2, *3 and *4). | 135 LARC patients (mixed, mainly white) | - Good risk patients: standard CRT using continuous infusional 5-FU at 225 mg/m2/d administered throughout radiation with no weekend breaks. - Poor risk patients: 5-FU/RT + weekly intravenous IRI at 50 mg/m.   RT: Total dose of 45-50.4Gy. | TRG  1, 2, 3-yrs RFS  1, 2, 3-yrs OS | DFS and ypT0 rates reaching 64.4% and 20% for good-risk (TYMS *2/*2, *2/*3, or *2/*4) and 64.5% and 42% for poor-risk (TYMS *3/*3 or *3/*4) patients, respectively. High rates of DFS and ypT0 were achieved among both risk groups when personalized treatment was based on TYMS genotype. |  | Tan et al., 2011 |
| TYMS-rs34743033 (2R/3R) (TSER*2 and *3), -rs2853542 (G>C). | 44 LARC patients (Asian, South Corea) | CT: 5-day infusion of 5-FU (425mg/m2 daily) + LV (20 mg/m2 daily) during weeks 1 and 5.  RT: Total dose of 45Gy over 5 weeks followed by a 5.4-Gy boost. | TRG (TRG1-2: good responders, TRG3-5 poor responders, Mandard system) | Low expression group (with a G>C SNP 2R/3RC or 3RC/3RC) compared to the high-expression group (without the SNP 2R/3RG, 3RC/3RG, or 3RG/3RG):   - exhibited a significantly greater tumor downstaging rate (*P*=0.001); - exhibited a significantly greater nodal downstaging rate (*P*=0.014). - had no significant difference in the TRG. |  | Hur et al., 2011 |
| TYMS-rs34743033 (2R/3R) (TSER*2 and *3), -rs2853542 (G>C), -rs16430 (1494del6). | 51 LARC patients (Caucasian, Hispanic) | CT: CAPE (825 mg/m2) twice daily during the entire RT period (n=7). 5-day infusion of 5-FU (1,000 mg/m2) during weeks 1 and 5 (n=20). Daily continuous infusion of 5-FU (225 mg/m2) during the entire RT period (n=24)  RT: Total dose of 45Gy  *63% of patients received also adj CT | TRG (pCR: no evidence of residual carcinoma; Rmic: presence of residual microfoci of carcinoma; Mandard and Dworak system) Responders: pCR + Rmic and non-responders: all others.  10-yrs recurrence (local and distant) PFS OS | 3R/3R genotype was associated with a higher response rate (pathological complete remission and microfoci residual tumor; 61 vs. 22% in *2/*2 and *2/*3; P = 0.013).  Multivariate analysis  3R/3R genotype was also an independent prognostic factor for better survival (*P*<0.05). |  | Paez et al., 2010 |
| TYMS-rs34743033 (2R/3R) (TSER*2 and *3), -rs2853542 (G>C), -rs16430 (1494del6) | 40 LARC patients (Caucasian) | CT: 120-h infusion of 1000 mg/m2 daily during weeks 1 and 5 of 5-FU  RT: Total dose of 50.4Gy | TRG (TRG0-1: non-responders; TRG2-4: responders) | All patients with TYMS 3'-UTR 6 bp/6 bp or 6bp/+6bp genotype (11/11) were responders as compared to only 20/26 (77%) of patients with TYMS3'-UTR +6bp/+6bp genotype (*P*=0.082). TS 3′-UTR 6 bp deletion slightly associated with tumor response. TS 5'-polymorphisms were not associated with neither tumour regression nor gene expression. |  | Stoehlmacher et al., 2008 |
| TYMS-rs34743033 (2R/3R); EGFR- rs712830; EGF-rs4444903. | 91 LARC | CT: UFT 300/mg/m2 daily + LV 22.5 mg/per day five days per week  RT: total dose of 65Gy | TRG (TRG1-2: major responders; TRG3-4: moderate responders) | A combination of biomarkers predictive for pCR was found:   - TYMS 2R/2R had a significantly higher rate of pCR compared with the 2/3 or 3/3 group. | - By combining EGF-rs4444903 and EGFR-rs712830 genotype analysis, a pCR rate of 64% in the combination group was found compared with an 87% risk of being a non-complete responder in the non-combination group. | Spindler et al., 2007 |
| TYMS-rs34743033 (2R/3R) (TSER*2 and *3) | 65 LARC patients (Caucasian, Hispanic) | 5-FU-based CRT | TRG  3-yr DFS | Patients with TYMS 3R/3R had a lower probability of downstaging than patients TYMS 2R/2R and 2R/3R (*P*=0.036; logistic regression). A trend toward improved 3-year DFS was detected in the 2R/2R and 2R/3R groups, compared with that in the 3R/3R group *(P*=0.17). |  | Villafranca et al., 2001 |
| **MTHFR** | | | | | | |
| TYMS-rs34743033, -rs16430, -rs34743033 together with -rs2853542); MTHFR-rs1801133, -rs1801131; ERCC1-rs11615, -rs3212986; ERCC2-rs13181; RAD51-rs1801320; XRCC1-rs25487, -rs1799782; XRCC3-rs861539; CCND1-rs9344; EGF-rs4444903; EGFR-rs712829, rs2227983. | **META-ANALYSIS for germ-line markers (14 performed in rectal cancer)**  1359 rectal patient  (Mainly Caucasian) | FPs-based (5-FU, CAPE) plus other drugs (platinum agents, cetuximab, IRI) CRT | TRG  (for the most part by Mandard and Dworak score system) | - MTHFR rs1801133-CC genotype resulted associated with major response under the random-effect model (OR:1.56, *P*=0.035). | - TYMS-rs34743033 2R/2R-2R/3R genotype: major response (OR:1.71, *P*=0.006; in Caucasian OR:1.74, *P*=0.008). - EGFR rs2227983-A allele was more frequent among patients with pCR (OR:0.21, *P*=0.005; random model). | Salnikova et al., 2016 |
| MTHFR-rs1801133 | 108 LARC patients | CT: 5-FU-based  RT: Total dose of 50.4Gy radiation over a period of 5 weeks. | TRG (TRG=0: responders; TRG=3: non-responders); 4-yrs recurrence (local and distant) | WT patients (CC) were 2.91 times more likely (291% increased benefit) to respond to nCRT (*P*=0.0150) and 3.25 times more likely (325% increased benefit) not to experience recurrence of the disease (*P*=0.0079) than patients with either the heterozygous (CT) or the homozygous mutation (TT) genotype. |  | Nikas et al., 2015 |
| MTHFR-rs1801133, -rs1801131; EGFR (HER-1)-rs45608036, -[rs2227983](http://www.ncbi.nlm.nih.gov/SNP/snp_ref.cgi?rs=2227983). | **META-ANALYSIS (11 studies, 8 performed in rectal cancer and 3 esophageal cancer)**  1337 cases  (839 cases for MTHFR rs1801133, 634 cases for MTHFR rs1801131, 340 cases for EGFR [rs2227983](http://www.ncbi.nlm.nih.gov/SNP/snp_ref.cgi?rs=2227983), 396 cases for EGFR rs45608036)  (80.8%, Caucasian; 9.6%, Asian; 9.6%, Mixed) | CT: FP (5-FU or CAPE) based ± other drugs (etoposide, cisplatin, CTX)  RT: Total dose of 36-50.5Gy. | TRG  (responders vs non-responders: TRG1–2 vs TRG3–5 or TRG1 vs TRG2–5, Mandard or system score) | MTHFR rs1801133: improved tumor response under the recessive model (CC vs. CT/TT) in overall analysis (OR:1.426, *P*=0.014), rectal cancer (OR:1.483, *P*=0.009), and TRG1–2 vs.3–5 group (OR:1.423, *P*=0.025). |  | Zhao et al., 2015 |
| 8 SNPs in 5 genes (MTHFR, SLC191A, SHMT1, DHFR, TYMS) | 745 LARC patients (mixed, mainly white) | Arm 1:   - bolus 5-FU in two 5-day cycles every 28 days before and after RT plus 5-FU via PVI 225 mg/m2/d during RT.   Arm 2:   - PVI 42 days before and 56 days after RT PVI.   Arm 3:   - bolus 5-FU+LV in two 5-day cycles before and after RT, plus bolus 5-FU+LV. | OS  DFS | No SNP was significantly associated with OS and DFS, only trends. |  | Ulrich et al., 2014 |
| 23 genes (APC, BRAF, CCND1, CTNNB1, DPYD, EGFR, ERCC1, P53, IL6, KRAS, mitD310, MTHFR, OGG1, OPRT, PARP1, PIK3CA, RAD23B, TLR2, TS, VEGF, XPA, XPC AND XPD). | 132 LARC patients (Mixed) | CT: continuous infusion of 5-FU 225 mg/m2 daily for 7 days.  RT: Total dose of 50.4Gy.  *44% patients received also adj CT. | TRG (yes/no pCR defined as the absence of any residual tumor in the rectal and dissected lymph nodes, AJCC system) | - None of the 27 patients with both p53 and KRAS mutations had a pCR. 51 out of 52 patients with both p53 and KRAS mutations or the rs603965-AA or MTHFR rs1801133-TT genotype were non-pCR. | - KRAS mutation (*P*=0.0145), CCND1 rs603965-AA genotype (*P*=0.0138), MTHFR rs1801133-TT genotype (*P*=0.0120): associated with non-pCR. | Garcia-Aguilar et al., 2011 |
| 25 SNPs in 16 genes  (XRCC1, XRCC3, ERCC1, ERCC2, RAD51, hMLH1, hMSH2, hOGG1, GSTP1, GSTT1, GSTM1, GSTA1 TYMS, MTHFR, ABCB1, ABCC2). | 238 LARC patients  (Caucasian, Italian) | CT: FP (mainly 5-FU) alone or in combination with other drugs (i.e., platinum derivatives, IRI or gefitinib). In 18% of patients raltitrexed was used in substitution to 5-FU.  RT: Total dose of 45–50.4Gy | TRG  (TRG≤2 vs TRG≥4, Mandard system) | - MTHFR rs1801133-T alleles (46.2 vs 57.3%, OR:0.48, *P*=0.034) was associated with lower chance to get a TRG≤2*.* - Other significant predictors by CART analysis were: ABCB1-rs1045642, MTHFR-rs1801133, ERCC1-rs3212986, ABCC2-rs717620, XRCC1-rs25487, XRCC3-rs1799794 together with patients’ gender. | - hOGG1 rs1052133-G (41.4 vs 55.4%, OR:0.46, P=0.024) was associated with lower chance to get a TRG≤2. - Other significant predictors by CART analysis were: ABCB1-rs1045642, MTHFR-rs1801133, ERCC1-rs3212986, ABCC2-rs717620, XRCC1-rs25487, XRCC3-rs1799794 together with patients’ gender. | Cecchin et al., 2011 |
| TYMS-rs34743033 (2R/3R) (TSER*2 and *3), -rs2853542 (G>C); MTHFR-rs1801133, - rs1801131. | 125 LARC patients (Caucasian) | CT: 5-FU based plus other drugs (platinum agents) RT: Total dose of 45–50.4Gy  *53% of patients received also adj CT | TRG (TRG 1–2; responders; TRG 3–5: non-responders, Mandard system) | - MTHFR rs1801133-T/rs1801131-A haplotype was found the only variable associated with tumor regression (*P*=0.004). - Patients not carrying the MTHFR rs1801133-T/rs1801131-A haplotype (OR:0.29, *P*=0.002) displayed a higher response rate than patients with the MTHFR rs1801133-T/rs1801131-A haplotype by multivariate analysis. |  | Terrazzino et al., 2006 |
| **DNA repair** | | | | | | |
| 66 SNPs in 10 genes (ERCC1, ERCC2, ERCC4, GSTP1, SOD2, XPA, XRRC1, XRCC3, MTHFR and TYMS). | 316 LARC patients | ACCORD-12 phase III trial randomly assigned to either   - RT total dose 45Gy + 800 mg/m2 CAPE twice daily 5 days per week - dose-intensified RT 50Gy over 5 weeks + 800 mg/m2 CAPE twice daily 5 days per week with CAPE 800 mg/m2 twice daily 5 days per week plus OXA 50 mg/m2 once weekly. | TRG (TRG0-1: non-responders; TRG2-3: responders, Dworak score)  Recurrence  5-yrs OS  5-yrs DFS | - In the CAPOX arm: four SNPs in ERCC2, XPA, MTHFR and ERCC1 were associated with tumor response. - In the overall population: interaction with treatment arm was significant for ERCC2 rs1799787 (*P*=0.05) and XPA rs3176683 (*P*=0.008), suggesting a predictive effect for response to CRT. All but XPA rs3176683 had a prognostic effect on tumor response.   Multivariate analysis  Interaction remained significant for XPA rs3176683 (OR:7.33, *P*=0.018) and the prognostic effect significant for ERCC2 rs1799787 (OR:0.55, *P*=0.027) and ERCC1 rs10412761 (OR:0.57, *P*= 0.042). Patients with the T/G haplotype of rs1799787 and rs10412761 had a 60% decrease in odds of response (*P*<0.001). | - In the CAPOX arm: rs7553194 SNP in MTHFR was associated with tumor response. | Boige et al., 2019 |
| RAD51-rs1801320 | 103 LARC patients  (Not specified) | Short-course RT with 5Gy over 5 days | OS  RFS  DMFS | - rs1801320-CC (vs GG or CC): longer OS (CC: 89.4 mo., CG:51.3 mo., GG: 56.3 mo.), lower local recurrences (CC:0%, CG: 23.5%, GG:76.5%) or distant metastases (CC:0%, CG:26.3%, GG:73.7%). - Female patients with radiosensitive phenotype (Ku70 expression ≤75.1% or rs1801320-CC genotype) had longer 5-yr OS (log-rank *P*=0.013, 93.3% vs 50.6%) than those with radioresistant phenotype (Ku70>75.1 % or rs1801320-GG/GC). |  | Gasinska et al., 2019 |
| 30 SNPs in 21 genes (XRCC1, XRCC3, MSH6, PARP1, OGG1, EXO1, ERCC2, ERCC1, MLH1, APEX1, MGMT, SOD2, ATM, TP53, MDM2, EGFR, EGF, VEGFA, GSTP1, MTHFR, TS). | 280 LARC patients^  (Caucasian)  *^ stratified in 3 groups according to RT dosage and association with OXA* | CT: FP alone (5-FU or CAPE) was prescribed with 50.4Gy or 55.0Gy dose; CAPE plus OXA with 50.4Gy dose.  RT: total dose of 50.4Gy or 55.0Gy | TRG  (TRG2–5 vs. TRG1, Mandard system) | Group 1 (n=94, OXA)  MDM2 rs2279744-G (OR:0.24, *P*=0.034): reduced chance of TRG1.  Group 2 (n=73, high RT dosage)  APE1 rs1130409-G: increased chance of TRG1 (additive model OR:3.20, *P*=0.025).  Group 3 (n=113, standard RT dosage)   - MSH6 rs3136228-G allele (OR:0.12, *P*=0.004) and MLH1 rs1799977-GG genotype (OR:0.23, *P*=0.026): lower rate of TRG1. - XRCC1 rs3213239-del (OR:3.24, *P*=0.020), VEGFA rs2010963-C (OR:3.14, *P*=0.022), ERCC1 rs3212986-T (OR:2.64, *P*=0.048) and MGMT rs12917-T (OR:3.31, *P*=0.042): higher rate of TRG1. | Group 1 (n=94, OXA)  MTHFR rs1801133-T (OR:3.49, *P*=0.019): increased chance of TRG1. | Dreussi et al., 2016a |
| XRCC1-rs25487, -rs179978; XRCC3-rs861539; ERCC1-rs11615; ERCC2-rs13181. | **META-ANALYSIS (5 studies)**  265 LARC patients  (Caucasian) | FPs-based (5-FU or CAPE) ± other drugs (i.e. CTX, OXA) in combination with RT | TRG  (TRG1-2 vs TRG3-5) | No polymorphism was associated with the response to RT-based multimodality treatment. |  | Guo et al., 2015 |
| 10 SNPs in 8 genes (XRCC1, ERCC1, ERCC2, EGFR, GSTP1, TYMS, MTHFR, DPYD). | 65LARC patients  (Caucasian) | CT: FPs-based (5-FU or CAPE)  RT: Total dose of 50.4Gy | TRG  (TRG1-2: good responders; TRG3-5: poor responders, Mandard system) | - XRCC1, rs25487-AA genotype (vs G-allele) associated with a better response rate (OR:7.93, *P*=0.036) when analyzed in tumor tissue DNA. | - TYMS high-expressing alleles (TSER*3G): better response rate (OR:2.19, *P*=0.022) by multivariate analysis. | Balboa et al., 2010 |
| XRCC1-rs25487, -rs1799782; XRCC3-rs861539. | 81 LARC patients  (Caucasian) | CT: 5-FU–based CRT  RT: Total dose of 50.4Gy | Response | In the XRCC1 rs25487-AG genotype group, 53% showed a minor response and 47% a major response. In contrast, in rs25487-AA/GG group, 78% showed minor response and only 22% major response (*P*=0.039). |  | Grimminger et al., 2010 |
| **Oxidative Stress / Detoxification** | | | | | | |
| GSTP-rs1695; XRCC1-rs25487. | 80 LARC patients (Caucasian) | CT: CAPE (825mg/mq twice daily) (n=48) or 5-FU (225 mg/mq daily continuous infusion) (n=32) on each day of RT.  RT: Total dose of 55Gy. | TRG (TRG0–3: non-complete responder; TRG4: complete responders)  5, 8-yrs OS  5, 8-yrs Cancer-specific survival | GSTP1 rs1695 may predict a higher rate of pCR after nCRT and a better outcome:   - AA genotype compared with AG+GG population presented a higher rate of pCR (26.6% vs 8.5%, *P*=0.04). - AA compared with AG genotype presented a higher rate of pCR (26.6% vs 6.8%; p=0.034). - AA+AG compared with GG patients presented a 5- and 8-year longer cancer-specific survival (*P*=0.014). |  | Nicosia et al., 2018 |
| 128 in 76 genes involved in DNA repair, apoptosis, proliferation or immune response. | 71 LARC patients  (Caucasian) | CT: CAPE-based ± OXA  RT: Total dose of 45-50Gy. | TRG  (TRG0-2: non-responders, TRG3-4: responders, Dworak system) | At multivariate analysis SOD2 rs4880-T (Val) (OR:0.19, *P*=0.005) allele was associated with lower chance of response. | IL-13 rs1800925-T (OR:0.14, P=0.0008) allele was associated with lower chance of response. | Ho-Pun-Cheung et al., 2011 |
| GSTM1 (deletion); GSTT1 (deletion); CAT-rs1001179; MnSOD-rs4880; MPO-rs2333227; NOS3-rs1799983. | 114 rectal cancer patients  (Caucasian) | RT containing protocols (n=39, neoadjuvant treatment, n=74 adjuvant/palliative treatment, n=1 both) | OS | NOS3 rs1799983-T (Asp) allele (vs GG) was associated with shorter OS (HR:2.10, *P*<0.05). *MPO* rs2333227-A allele (vs GG) was associated with longer OS (HR:0.44, *P*<0.05), but there was no allele dose effect. |  | Funke et al., 2009 |
| **Cellular proliferation / EGFR pathway** | | | | | | |
| 16 TagSNPs in 5 genes (PIK3CA, PTEN, AKT1, AKT2, FRAP1[encodes mTOR]). | 97 LARC patients  (Chinese) | CT: XELOX  RT: Total dose of 50Gy. | TRG (TRG1: poor response vs TRG:2-4 significant response, Dworak system)  DSF  OS | - PTEN rs12569998-G allele (vs TT): increased tumor response rate (adjusted OR:2.909, P=0.027). - AKT2 rs8100018-C allele (vs. GG): decreased recurrence risk (adjusted HR:0.414, *P*=0.029) and longer 5-yr DFS rate (79.2% vs. 62.3%, *P*=0.038). |  | Peng et al., 2018 |
| 28 SNPs in the EGFR pathway (EGFR, EGF, AREG, EREG), TYMS and DNA repair (ERCC1, XPD, XRCC1) genes. | 84 LARC patients  (Caucasian, Hispanic) | CT: CAPE-based  RT: Total dose of 45Gy | TRG  (Mandard system) | - AREG rs11942466 (CC: 35.7%, CA: 19.4%, AA: 0%; P=0.0018, co-dominant model), ERCC1-rs11615 (TT: 30.6%, TC:16.2%, CC:0%; *P*=0.0235, co-dominant model), ERCC1-rs3212986 (GG:29.2%, GT:9.7%, TT:0%; *P*=0.0096, additive model): associated with the pCR rate also by multivariate analysis. - AREG rs11942466 resulted the most discriminating factor by CART analysis. |  | Sebio et al., 2015 |
| Let-7 microRNA-binding site, LCS6-rs61764370 in the 3’UTR of Kras. | 155 LARC patients  (Caucasian) | CAPOX followed by CAPE-based CRT, surgery and adjuvant CAPOX ± CTX (EXPERT-C trial) | Response  PFS | - LCS6 rs61764370-G allele: higher complete response rate after nCRT (28.1% vs 10.6%, *P*=0.020), better 5-yr PFS (77.4% vs 64.5%, HR:0.56, *P*=0.152] and OS rates (80.3% vs 71.9%, HR:0.59, *P*=0.234). - The negative prognostic effect associated with KRAS mutation appeared to be stronger in patients with the rs61764370-TT genotype (PFS, HR:1.70, *P*=0.078; OS, HR:1.79, *P*=0.082) compared with those with the G-allele genotype (PFS, HR:1.33, *P*=0.713; OS HR:1.01, *P*=0.995). |  | Sclafani et al., 2015 |
| P73-2273953, -1801173 (G4C14 → A4T14). | 138 rectal cancer patients  (Caucasian) | RT: Total dose of 25Gy (n=65)  Surgery alone (n=73) | DFS | In the RT group   - AT (GC/AT + AT/AT) allele: stronger expression of [p53](https://www.sciencedirect.com/topics/medicine-and-dentistry/protein-p53) (*P=*0.001) and [survivin](https://www.sciencedirect.com/topics/medicine-and-dentistry/survivin) protein (*P=*0.03) than GC/GC genotype. - GC/GC genotype, along with negative p53 and weak survivin expression was associated with better DFS than other genotype/phenotype combinations in multivariate analysis (RR:7.63, *P=*0.01). |  | Loof et al., 2009 |
| CCDN1-rs9344 | 70 LARC patients  (Caucasian) | RT: Total dose of 45-60Gy | TRG  (TRG0-1: non-responders, TRG2-4: responders, Dworak system)  Local recurrence  RFS  OS | - rs9344-AA genotype: improved tumor regression (good responders, AA:90%, AG:42.5%, GG:60%, *P*=0.022). At multivariate analysis, rs9344-AA correlated with increased response (HR:10.0, *P*=0.034) and was the only independent predictor. - rs9344-A allele (vs GG): lower risk of local failure (5.9% vs 26.3%, *P=*0.017). At multivariate analysis, GG genotype was confirmed as predictors of increased risk of developing a local recurrence (HR:5.6, *P*=0.020). - Combination of rs9344 with post-therapeutic lymph node status allowed the elaboration of a prognostic index, which accurately distinguished subgroups of patients with predictable RFS (*P*=0.003) and OS (*P*=0.044). |  | Ho-Pun-Cheung et al., 2007 |
| EGFR (HER-1) Sp1 -216 G/T (rs712830) | 77 LARC patients  (Caucasian) | CT: UFT  RT: Total dose of 65Gy | TRG  (TRG1-2: major response; TRG3-4: moderate response; TRG5: no response) | EGFR rs712830-GG genotype (vs T-allele): lower response rate (34% vs 65%, *P*=0.023). |  | Spindler et al., 2006 |
| EGFR (HER-1)-rs45608036, -[rs2227983](http://www.ncbi.nlm.nih.gov/SNP/snp_ref.cgi?rs=2227983). | 59 LARC patients  (61% Caucasian, 22% Hispanic, 12% Asian 5% African-American) | CT: 5-FU-based  RT: Total dose of 50.4-54Gy (n=16, neoadjuvant treatment, n=43 adjuvant treatment) | Time to Local Recurrence | - EGFR [rs2227983](http://www.ncbi.nlm.nih.gov/SNP/snp_ref.cgi?rs=2227983)-GG (Arg/Arg) genotype or lower number of rs45608036-CA repeats (both alleles <20): non-significant increased risk of local recurrence (log-rank test, *P*=0.24 and 0.31, respectively). - Patients carrying both [rs2227983](http://www.ncbi.nlm.nih.gov/SNP/snp_ref.cgi?rs=2227983)-G (Arg) and rs45608036 <20 CA repeats showed the highest risk for local recurrence *(*log-rank test, *P*=0.05). |  | Zhang et al., 2005 |
| **Microenvironment** | | | | | | |
| 192 SNPs in 34 genes involved in the regulation of the immune response signaling network (CD276 (B7-H3), CXCR7, FAS, FOXO3, FOXP3, IFNG, IFNGR1, IFNGR2, IL15RA, IL17A, IL17F, IL2RA, IL2RB, IL2RG, IL8, MIF, MMP3, PRDM1, SMAD3, SMAD4, STAT3, STAT5A, STAT5B, STAT6, TGFBR1, TGFBR2, TIMP1, TIRAP, TLR10, TLR3, TLR4, TLR6, VEGFA, WNT5A) | 370 LARC patients (233, test set; 137 validation set)  (Caucasian) | CT: FPs (5-FU or CAPE) based ± OXA  RT: total dose of ≤50.40Gy (72.7%) or >50.40Gy (27.3%) | 2-yr DFS  5-yr MFS  5-yr DFS  10-yr OS | - IL17F rs641701-C (test, HR:4.62, *P*=0.002; validation, HR:3.62, *P*=0.044) and IL17F-rs9463772-A (test, HR:3.12, *P*=0.006; validation, HR:2.78, *P*=0.088): worse 2-yr DFS in both cohort.   Pooled population:   - *IL17F* rs641701-C: worse 5-yr DFS (HR=1.84, *P*=0.035; log-rank *P*=0.055), 5-yrMFS (HR:2.11, *P*=0.023; log-rank *P*=0.020) and 10-yr OS (HR:2.66, *P*=0.001; log-rank *P*=0.003). - IL17F rs9463772-A: worse 5-yrMFS (HR:1.82, *P*=0.031; log-rank *P*=0.040) and 10-yr OS (HR:2.00, *P*=0.009; log-rank *P*=0.012)   TRG subgroup analysis: TRG1, n=109, subgroup:   - IL17F rs641701-C: worse 5-yr DFS (HR: 4.25, *P*=0.014) and 10-yr OS (HR:5.14, *P*=0.003); - IL17F-9463772-A: a trend towards worse and 10-yr OS (HR:2.58, *P*=0.093).   *TRG/genotype stratified subgroups:*  The combination of TRG with IL17F-rs641701 and IL17F-rs9463772 genotype allowed the identification of subgroups of patients with differential prognosis in term of both 5yrDFS (HR:11.29, *P*<0.001; HR:5.86, *P*=0.001, respectively) and 10yrOS (HR:7.07, *P*=0.005; HR:6.05, *P*=0.002, respectively). |  | Cecchin et al., 2020 |
| Whole-exome sequencing | 97 LARC patients  (30, discovery cohort; 67 validation cohort)  (Korean) | CT: 5-FU-based  RT: Total dose of 45Gy | TRG (yes/no pCR defined as the absence of any residual tumor in the rectal and dissected lymph nodes, AJCC system)  RFS  OS | DLC1-rs3816748 GC/CC (vs GG) (*P*=0.032), DNAH14 rs3105571-AC/CC (vs AA) (*P*=0.009), and RAET1 rs912565-TT (vs TC/CC) (*P*<0.0001) BCL2L10 rs2231292-CC (vs AA/AC) (*P*=0.036) and ITIH5 rs3824658-TC/TT (vs CC) (*P*=0.003): higher pCR rate. |  | Lee et al., 2018 |
| 114 microRNA-related TagSNPs | 265 LARC patients  (Caucasian) | CT:   - FP alone (5-FU or CAPE) was prescribed with 50.4Gy or 55.0Gy dose; - CAPE plus OXA with 50.4Gy dose.   RT: total dose of 50.4Gy (n=188) or 55.0Gy (n=77) | TRG  (TRG2-5 vs TRG1, Mandard system)  DFS | - SMAD3-rs744910A>G (OR:0.45, *P*=0.0153), SMAD3-rs745103A>G (OR:0.48, *P*=0.0471), and TRBP-rs6088619A>G (OR:0.39, *P*=0.0125): increased chance of TRG1. - DROSHA-rs10719C>T (OR:1.87, *P*=0.0274) and SMAD3-rs17228212T>C (OR:2.01, *P*=0.0049): a higher risk of TRG2-5. - *TRBP-*rs6088619-G allele: trend for longer DFS (HR:0.24, *P*=0.0706).   CART analysis  Patients with a specific combination of SMAD3-rs744910 and TRBP-rs6088619 genotypes benefited from a longer interval time between the end of CRT and surgery. |  | Dreussi et al., 2016b |
| miR-608- rs4919510 | 155 LARC patients  (Caucasian) | CAPOX (four cycles) followed by CAPE-based CRT  (EXPERT-C trial) | PFS  OS | rs4919510-CC genotype (vs G-allele) was associated with worse 5-yr PFS (54.6% vs 82.0%, HR:0.13, *P*=0.010) and 5-yr OS (60.7% vs 82.1%, HR:0.38, *P*=0.033). |  | Sclafani et al., 2016 |
| IL13- rs1800925 | 46 LARC patients  (Chinese) | CT: XELOX or modified FOLFOX6 RT: Total dose of 46Gy | TRG  (TRG0-2: poor responders, TRG3-4: good responders, Dworak system)  PFS  OS | No significant associations were found. |  | Xiao et al., 2016 |
| NFKB1-rs28362491; PTGS1-rs1213266, -rs5789; PTGS2-rs5275; IL1B-rs16944, -rs1143627. | 159 LARC patients  (Hispanic) | CT: CAPE-based  RT: Total dose of 50.4Gy | TRG  (ypT3-4 and/or pN+: poor response vs ypT0N0: complete or ypT1-2N0; intermediate/partial response, AJCC scale)  DFS  OS | - NFKB1 rs28362491-DEL/DEL genotype (vs INS-allele): increased rate of pathologic response (OR:6.39, *P*=0.03), a trend for longer DFS (log-rank test, *P*=0.096) and OS (log-rank test, *P*=0.049), which was not significant in a multivariate analysis. - by haplotype analysis, rs28362491-DEL/rs1143627-A/rs1213266-G/rs5789-C/rs5275-A/rs16944-G was associated with higher response rate (OR:8.86, *P*=0.034) than the reference haplotype (rs28362491-INS/rs1143627-A/rs1213266-G/rs5789-C/rs5275-A/rs16944-G). |  | Dzhugashvili et al., 2014 |
| Genome-wide SNP array | 113 LARC patients  (Korean) | CT: FPs-based (5-FU or CAPE)  RT: total dose of 45Gy followed by a boost to the primary tumor of 5.4Gy over 5 weeks. | TRG (TRG1-3 vs TRG4-5, Mandard system) | - CORO2A rs1985859-C reference allele (vs T-allele): better response (TRG1-3) (*P*=0.01). Down-regulation of *CORO2A (*linked to the T-allele) was associated with reduced early apoptosis by 27% (*P*=0.048) and 39% (*P*=0.023) in vitro. Reduced radiosensitivity was confirmed by colony-forming assays in the 2 CRC cells (*P*=0.034 and 0.015, respectively). - FAM101A rs7955740: not associated with response. Down-regulation of *FAM101A,* linked to the minor G-allele, reduced early apoptosis and enhanced colony formation in vitro. |  | Kim et al., 2013 |
| HIF1A- rs11549465, rs11549467, rs2057482. | 263 LARC patients  (65, test cohort; 198 validation cohort)  (Caucasian) | Test cohort: UFT/isovorin and RT at total dose of 60Gy plus 5Gy of brachytherapy.  Validation cohort: UFT/LV and RT at total dose of 50.4Gy ± 10Gy of brachytherapy. | TRG  (Endpoint, pCR defined as TRG 1 plus no lymph node metastases, Mandard system) | HIF1A rs2057482-CC genotype was marginally associated with a higher rate of pCR (*P*=0.05) in the test cohort, while with a poor tumour response (*P*=0.03) in the validation cohort. |  | Havelund et al., 2012 |
| 21 SNPs in 18 genes (TYMS, GSTM1, GSTT1, GSTP1, ERCC1, XRCC3, APE1, RAD51, IL-8, IL-10, TGFB1, MMP-3, VEGFA, FGFR4, PTGS2, ICAM-1 TP53, CCND1). | 90 LARC patients  (68% Caucasian, 32% others) | CT: 5-FU-based  RT: Total dose of 50.4-54.0Gy (n=23)  *Adjuvant 5-FU plus radiation (n=67) | Recurrence rate | - IL-8 rs4073-AA genotype: increased risk of recurrence (recurrence rate, AA:88%, AT:57%, TT:38%, *P*=0.029). - PTGS2 rs20417-C: non-significant decreased risk of recurrence (recurrence rate, GG:56%, GC:29%, *P*=0.081). - IL-8-rs4073, ICAM-1-rs5498, TGFB1-rs1800470, FGFR4-rs351855 highlighted by CART analysis as markers of recurrence. | - GSTP1 rs1695-GG (Val/Val) genotype (recurrence rate, GG:83%, GA, 42%, AA:58%, *P*=0.089) and TGFB1 rs1800470-C allele (recurrence rate, TT:37%, CT: 60%, CC:57%, *P*=0.12): increased risk of recurrence. | Gordon et al., 2006 |

Abbreviations: 5-FU, 5-fluorouracil; Adj, adjusted; AJCC, American Joint Committee on Cancer Tumor; CAPE, capecitabine; CAPOX, capecitabine and oxaliplatin; CART, classification and regression tree; CRC, colorectal cancer; CRM, circumferential rectal margin; CRT, chemoradiotherapy; CT, chemotherapy; CTX, cetuximab; del., deletion; DFS, disease-free survival; DMFS, distant metastasis-free survival; FOLFOX, folinic acid, fluorouracil and oxaliplatin; FPs, fluoropyrimidines; HR, hazard ratio; Ins., insertion; IRI, irinotecan; LV, leucovorin; MFS, metastases-free survival; mo., months; OR, odd ratio; OXA, oxaliplatin; pCR, complete patho­logical response; PFS, progression-free survival; PVI, protracted venous infusion; RFS, recurrence-free survival; RR, risk ratio; OS, overall survival; RR, response rate; RT, radiotherapy; SNP, single nucleotide polymorphism; TagSNPs, tagging polymorphisms; TRG, tumor regression grade; WT, wild-type; XELOX, oxaliplatin and capecitabine; yr, year; UFT, Tegafur and Uracil (Uftoral).
